# A Systematic Review With Network Meta-Analysis of the Available Biologic Therapies for Psoriatic Disease Domains

**DOI:** 10.3389/fmed.2020.618163

**Published:** 2021-01-15

**Authors:** Tiago Torres, Anabela Barcelos, Paulo Filipe, João Eurico Fonseca

**Affiliations:** ^1^Department of Dermatology, Centro Hospitalar Universitário do Porto, Porto, Portugal; ^2^Multidisciplinar Medical Research Unit, Instituto de Ciências Biomédicas Abel Salazar, University of Porto, Porto, Portugal; ^3^Rheumatology Department, Centro Hospitalar do Baixo Vouga, Aveiro, Portugal; ^4^NOVA National School of Public Health, Public Health Research Centre, Universidade NOVA de Lisboa – Portugal, Lisbon, Portugal; ^5^Comprehensive Health Research Center (CHRC), Universidade NOVA de Lisboa – Portugal, Lisbon, Portugal; ^6^Serviço de Dermatologia e Venereologia, Hospital de Santa Maria, Centro Hospitalar Universitário Lisboa Norte, Lisbon, Portugal; ^7^Unidade de Investigação em Dermatologia, Instituto de Medicina Molecular João Lobo Antunes, Faculdade de Medicina, Universidade de Lisboa, Lisbon, Portugal; ^8^Clínica Universitária de Dermatologia, Faculdade de Medicina da Universidade de Lisboa, Lisbon, Portugal; ^9^Serviço de Reumatologia e Doenças Ósseas Metabólicas, Hospital de Santa Maria, Centro Hospitalar Universitário Lisboa Norte, Lisbon, Portugal; ^10^Unidade de Investigação em Reumatologia, Instituto de Medicina Molecular João Lobo Antunes, Faculdade de Medicina, Universidade de Lisboa, Lisbon, Portugal

**Keywords:** psoriasis, psoriatic arthritis, psoriatic disease, biologic therapy, systematic review, network meta-analysis

## Abstract

**Introduction:** Several new treatments have been developed for psoriatic disease, an inflammatory condition that involves skin and joints. Notwithstanding, few studies have made direct comparisons between treatments and therefore it is difficult to select the ideal treatment for an individual patient. The aim of this systematic review with network meta-analysis (NMA) was to analyze available and approved biologic therapies for each domain of psoriatic disease: skin, peripheral arthritis, axial arthritis, enthesitis, dactylitis, and nail involvement.

**Methods:** Data from randomized clinical trials (RCTs) were included. A systematic review was performed using the MEDLINE database (July 2020) using PICO criteria. Bayesian NMA was conducted to compare the clinical efficacy of biological therapy in terms of the American College of Rheumatology criteria (ACR, 24 weeks) and Psoriasis Area and Severity Index (PASI, 10–16 weeks).

**Results:** Fifty-four RCTs were included in the systematic review. Due to the design of the RCTs, namely, outcomes and time points, network meta-analysis was performed for skin and peripheral arthritis domains. For the skin domain, 30 studies reporting PASI100 were included. The peripheral arthritis domain was analyzed through ACR70 in 12 studies. From the therapies approved for both domains, secukinumab and ixekizumab were the ones with the highest probability of reaching the proposed outcomes. There is a lack of outcome uniformization in the dactylitis, enthesitis, and nail domains, and therefore, an objective comparison of the studies was not feasible. Nevertheless, secukinumab was the treatment with the best compromise between the number of studies in each domain and the results obtained in the different outcomes.

**Conclusion:** Secukinumab and ixekizumab were the treatments with the highest probability of reaching both PASI100 and ACR70 outcomes. Due to the lack of a standard evaluation of outcomes of the other psoriatic disease domains, a network meta-analysis for all the domains was not possible to perform.

## Introduction

Psoriasis (PsO) affects 1–3% of the world population. Psoriatic arthritis (PsA) occurs in a third of the patients with PsO. These two conditions share clinical, genetic, and pathogenic factors and can be considered a single entity—psoriatic disease (PsD) (1–3).

PsD involves chronic inflammation of the skin, nails, and joints (arthritis, enthesitis, dactylitis, and spondylitis) (4). Autoimmune mechanisms are involved in PsA pathogenesis, and this is ultimately related with the systemic nature of the disease and raised the concept of a Systemic Psoriatic Disease. This fact highlights the heterogeneity of the disease and the need for optimizing its management (5).

Optimal management of PsD requires early diagnosis, monitoring of the disease activity, and treatment with effective and safe therapies. Over the last 20 years, targeted therapies emerged in the treatment of PsD, namely, biologic agents such as tumor necrosis factor inhibitors (TNFi), IL-17 inhibitors (IL-17i), and IL-12/23 inhibitors (IL-12/23i), and small molecules, such as Janus Kinase (JAK) or phosphodiesterase 4 (PDE4) inhibitors (6).

The Group for Research and Assessment of Psoriasis and Psoriatic Arthritis (GRAPPA) is a global association of more than 500 rheumatologists, dermatologists, and patient research partners that publish treatment recommendations for PsD (2). The treatment of six domains—peripheral arthritis, axial disease, enthesitis, dactylitis, skin disease, and nail disease—are included in the recommendations directed to anyone involved in the treatment of patients with PsD (2). Based on these recommendations, we performed a systematic review and network meta-analyses assessing the main results of randomized clinical trials (RCT) including biologic therapies in the treatment of patients with PsD.

## Methods

### Literature Search

A literature search according to the Population, Intervention, Comparator, Outcomes (PICO) framework was performed establishing criteria for study eligibility. The population was defined as adult (≥18 years) patients with the PsD (PsO and/or PsA) and the intervention as any biologic therapy: adalimumab (ADA), etanercept (ETN), infliximab (IFX), golimumab (GOL), certolizumab (CZP), ustekinumab (UST), secukinumab (SEC), ixekizumab (IXE), guselkumab (GUS), brodalumab (BRD), risankizumab (RIS), and tildrakizumab (TIL), in all formulations and treatment durations. The comparator was the same drug (different dose or regimen), any different drug, or placebo. Outcomes considered were American College of Rheumatology (ACR) or Psoriasis Area Severity Index (PASI) or dactylitis assessment or enthesitis assessment or nail psoriasis assessment. The MEDLINE database search was performed on 1 July 2020, with the filters “Humans,” “Clinical Trials,” “Phase III,” and “English,” with no date limits. In line with the GRAPPA and EULAR recommendations, we did not include abatacept in this systematic review. In addition, as this systematic review was focused only on biologic treatments apremilast and tofacitinib were not analyzed.

### Statistics and Network Meta-Analyses

Network meta-analyses (NMA) were carried out using the web application CINeMA 1.9.0 (Confidence in Network Meta-Analysis) from Cochrane (7). This application is based on a described methodological framework that considers six domains: within-study bias, reporting bias, indirectness, imprecision, heterogeneity, and incoherence (8). NMAs based on the Bayesian framework using the fixed-effects model were performed to pool all the direct and indirect evidence together. Odds ratio (OR) with 95% credible intervals (CrI) was used to evaluate comparisons. Only comparisons showing high confidence in the six domains were considered for the results.

### Assessment of Bias

Assessment of bias was performed using the latest version of RoB2—Cochrane (9).

## Results

**Figure 1:**
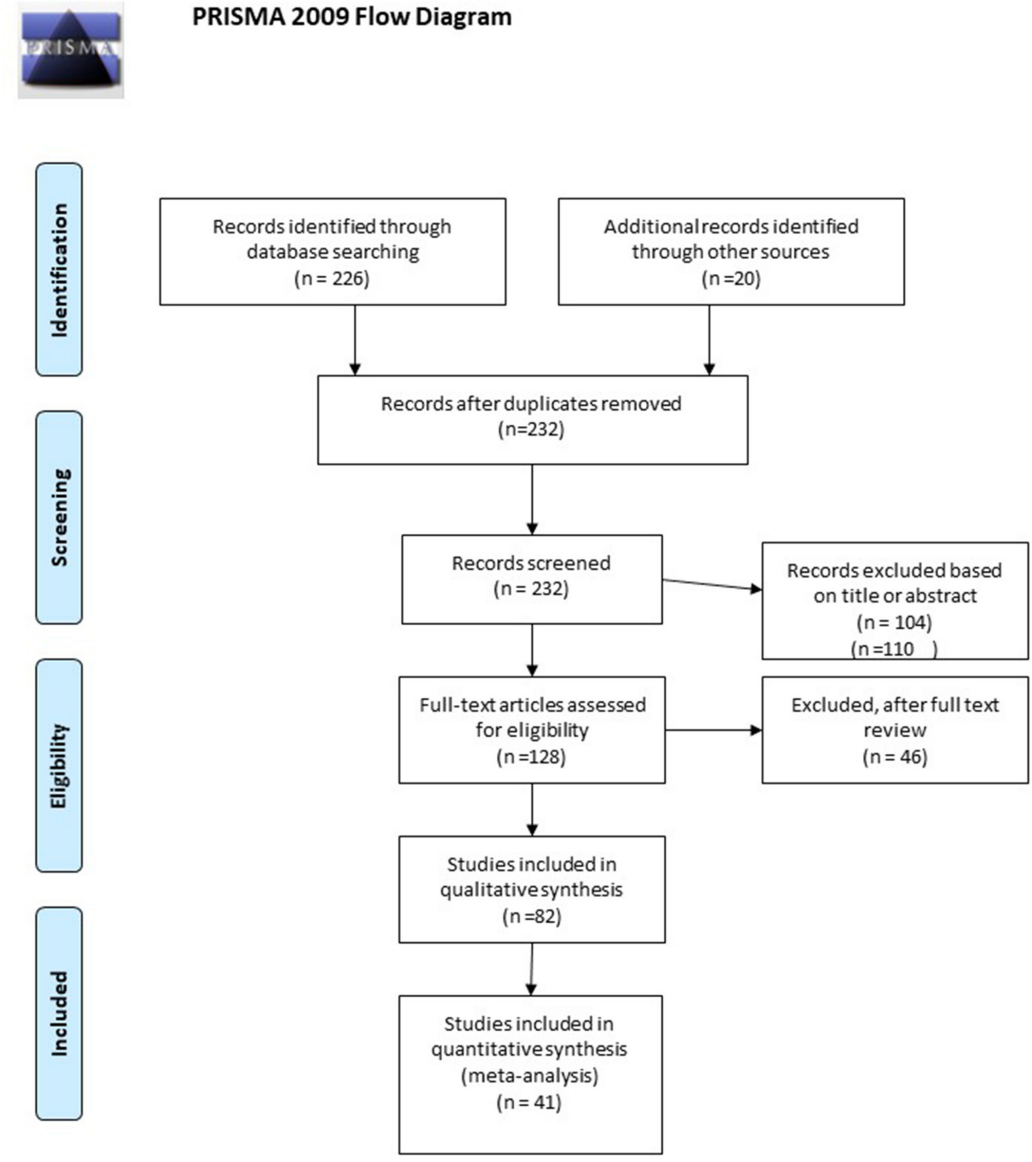
PRIMA flow diagram. Adapted from (10).

**Figure 2:**
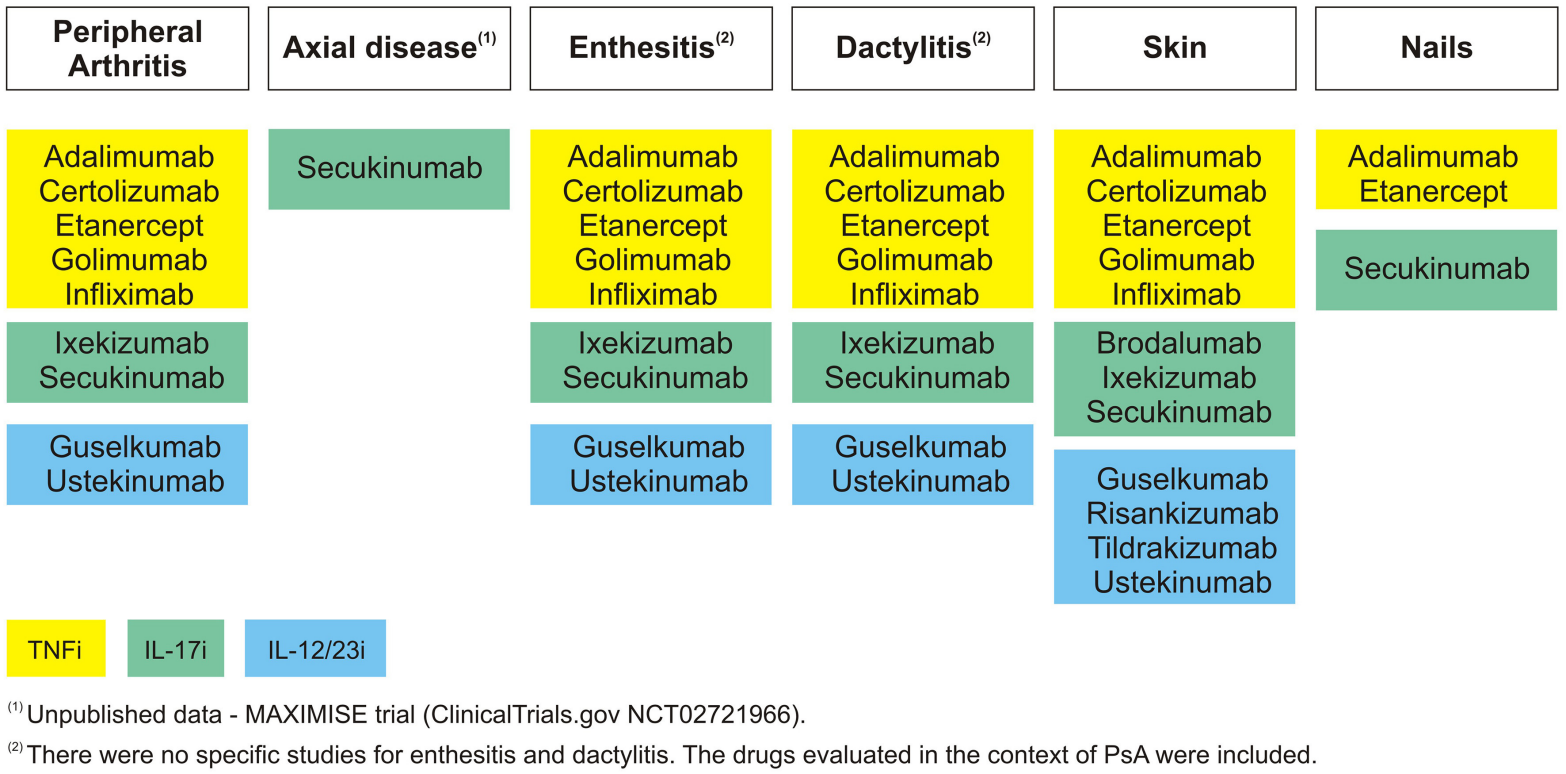
GRAPPA domains—evaluated therapies.

A detailed flowchart with the results of the literature review is shown in Figure 1. Out of the 232 references retrieved, 82 studies were selected for data (1, 11–57). For NMAs, only studies reporting ACR20, ACR50, ACR70 (peripheral arthritis domain), PASI75, PASI90, or PASI100 (skin domain) were included. For the peripheral arthritis domain, only 24 weeks were included. For the skin domain, results between 10 and 16 weeks were considered. Moreover, the doses of the drugs for the systematic review and NMAs, for the peripheral arthritis and skin domains, were the ones approved by the regulatory authorities. The studies included in the NMAs are identified in Table 1. Extension studies are specified in Table 2 (48, 58–84). In Figure 2 the drugs that have been studied specifically for each domain of PsD were included.

**Table 1:** RCT included in the systematic review and NMA, focusing on the outcomes of GRAPPA domains.

**Study**	**Enrolled patients**						**NMA**
	**Author**	**Year**	***N***	**Drug**	**Dosage**	**Outcomes**	
IMPACT 2 (1)	Antoni	2005	200	IFX	5 mg/kg	①②③④	YES
				PLB		⑤⑥⑦⑧	
ADEPT (11)	Mease	2005	313	ADA	40 mg	①②③	YES
				PLB		④⑤⑥	
(12)	Reich	2005	378	IFX	5 mg/kg	④⑤	YES
				PLB		⑥⑩	
(13)	Genovese	2007	100	ADA	40 mg	①②③	NO
				PLB		⑦⑨	
(14)	Tyring	2007	618	ETN	50 mg	④⑤⑥	YES
				PLB			
PHOENIX 1 (15)	Leonardi	2008	766	UST	45 mg	④⑤⑥	YES
				UST	90 mg		
				PLB			
PHOENIX 1 (16)	Papp	2008	1,230	UST	45 mg	④⑤⑥	YES
				UST	90 mg		
				PLB			
(17)	Rich	2008	378	IFX	5 mg/kg	⑩	NO
				PLB			
(18)	Kavanaugh	2009	405	GOL	50 mg	①②③ ④⑤⑥	YES
				GOL	100 mg	⑧⑨⑩	
				PLB			
(19)	Barker	2011	868	IFX	5 mg/kg	④⑤⑥	YES
				MTX	15 mg		
(20)	Gottlieb	2011	347	BRK	200 mg	⑤⑥⑦	YES
				ETN	50 mg		
				PLB			
(21)	Strober	2011	350	BRK	200 mg	⑤⑥⑦	YES
				ETN	50 mg		
				PLB			
RESPOND (22)	Baranauskaite	2012	115	IFX + MTX	5 mg/kg	①②③	NO
				MTX	15 mg	⑤⑧⑨	
(23)	Gottlieb	2012	478	MTX + ETN	15 mg + 50 mg	④⑤⑥	NO
PSUMMIT 1 (24)	McInnes	2013	615	UST	45 mg	①②③	YES
				UST	90 mg	⑥⑧⑨	
				PLB			
ERASURE (25)	Langley	2014	738	SEC	150 mg	⑤⑥⑦	YES
				SEC	300 mg		
				PLB			
FIXTURE (25)	Langley	2014	1,306	SEC	150 mg	⑤⑥⑦	YES
				SEC	300 mg		
				ETN	50 mg		
				PLB			
RAPID-PsA (26)	Mease	2014	409	CZP	200 mg	①②③ ④⑤⑥	YES
				CZP	400 mg	⑧⑨⑩	
				PLB			
PHOENIX 1 (27)	Rich	2014	766	UST	45 mg	④⑤	YES
				UST	90 mg	⑥⑩	
				PLB			
PSUMMIT 2 (28)	Ritchlin	2014	312	UST	45 mg	①②③	YES
				UST	90 mg	⑤⑧⑨	
				PLB			
UNOCOVER 2 (29)	Griffiths	2015	1,224	IXE	80 mg 2 w	⑤⑥⑦	YES
				IXE	80 mg 4 w		
				ETN	50 mg		
				PLB			
UNOCOVER 3 (29)	Griffiths	2015	1,346	IXE	80 mg 2 w		YES
				IXE	80 mg 4 w	⑤⑥⑦	
				ETN	50 mg		
				PLB			
AMAGINE-2 (30)	Lebwohl	2015	1,831	BRD	140 mg	⑤⑦	YES
				BRD	210 mg		
				UST	45/95 mg		
				PLB			
AMAGINE-3 (30)	Lebwohl	2015	1,881	BRD	140 mg	⑤⑦	YES
				BRD	210 mg		
				UST	45/95 mg		
				PLB			
FUTURE 2 (31)	McInnes	2015	397	SEC	75 mg	①②⑤	YES
				SEC	150 mg	⑥⑧⑨	
				SEC	300 mg		
				PLB			
(32)	Mease	2015	606	SEC	10 mg/kg	①②⑤	YES
				SEC	75 mg	⑥⑧⑨	
				SEC	150 mg		
				PLB			
CLEAR (33)	Thaçi	2015	676	SEC	300 mg	⑤⑥⑦	YES
				UST	45/90 mg		
BELIEVE (34)	Thaçi	2015	730	ADA	40 mg	⑩	NO
				PLB			
AMAGINE-1 (35)	Papp	2016	661	BRD	140 mg	⑤⑥⑦	YES
				BRD	210 mg		
				PLB			
VOYAGE 1 (36)	Blauvelt	2017	837	GUS	100 mg	⑤⑥⑦⑩	YES
				ADA	40 mg		
				PLB			
SPIRIT-P1 (37)	Mease	2017	417	IXE	80 mg 2 w	①②⑤	YES
				IXE	80 mg 4 w	⑥⑦⑧	
				ADA	40 mg	⑨⑩	
				PLB			
SPIRIP-P2 (38)	Nash	2017	363	IXE	80 mg 2 w	①②③	YES
				IXE	80 mg 4 w	⑤⑥⑦	
				PLB		⑧⑨⑩	
reSURFACE 1 (39)	Reich	2017	772	TIL	100 mg	⑤⑥⑦	YES
				TIL	200 mg		
				PLB			
reSURFACE 2 (39)	Reich	2017	1,090	TIL	100 mg	⑤⑥⑦	YES
				TIL	200 mg		
				ETN	50 mg		
				PLB			
IXORA-S (40)	Reich	2017	302	IXE	80 mg	⑤⑥⑦	YES
				UST	45/90 mg		
CLARITY (41)	Bagel	2018	1102	SEC	300 mg	⑤⑥⑦	YES
				UST	45/90 mg		
(42)	Elewski	2018	217	ADA	40 mg	⑩	NO
				PLB			
UltIMMa-1 (43)	Gordon	2018	506	RIS	150 mg	⑤⑥⑦	YES
				UST	45/90 mg		
				PLB			
UltIMMa-2 (43)	Gordon	2018	491	RIS	150 mg	⑤⑥⑦	YES
				UST	45/90 mg		
				PLB			
CIMPASI-1 (44)	Gottlieb	2018	234	CZP	200 mg	⑥⑦	YES
				CZP	400 mg		
				PLB			
CIMPASI-2 (44)	Gottlieb	2018	227	CZP	200 mg	⑥⑦	YES
				CZP	400 mg		
				PLB			
CIMPACT (45)	Lebwohl	2018	559	CZP	200 mg	⑤⑥	YES
				CZP	400 mg		
				ETN	50 mg		
				PLB			
TRANSFIGURE (46)	Reich	2018	198	SEC	150 mg	⑤⑥	YES
				SEC	300 mg	⑦⑩	
				PLB			
FUTURE 5 (47)	Mease	2018	774	SEC	150 mg	①②③	YES
				SEC	300 mg	⑤⑦⑧	
				PLB			
SustaIMM (48)	Ohtsuki	2019	171	RIS	75 mg	⑤⑥⑦	YES
				RIS	150 mg		
				PLB			
ECLIPSA (50)	Araujo	2019	47	UST	45/90 mg	⑨	NO
				TNFi			
IMMvent (52)	Reich	2019	605	RIS	150 mg	⑤⑥⑦	YES
				ADA	40 mg		
ECLIPSE (53)	Reich	2019	1048	GUS	100 mg	⑤⑥⑦	YES
				SEC	300 mg		
DISCOVER-2 (49)	Mease	2020	741	GUS	100 mg	①②③⑤	NO
				PLB		⑥⑦⑧⑨	
SPIRIT H2H (51)	Mease	2020	566	ADA	40 mg	①②③⑤	YES
				IXE	80 mg	⑥⑧⑨⑩	
DISCOVER-1 (54)	Deodhar	2020	624	GUS	100 mg	①②③⑤	NO
				PLB		⑥⑦⑧⑨	
EXCEED (55)	McInnes	2020	853	SEC	300 mg	①②③⑤	NO
				ADA	40 mg	⑥⑦⑧⑨	
ORION (56)	Ferris	2020	78	GUS	100 mg	⑤⑥⑦	YES
				PLB			
IMMerge (57)	Warren	2020	327	RIS	150 mg	⑤⑥⑦	YES
				SEC	300 mg		

*N, number; NMA, network meta-analysis; GRAPPA, Group for Research and Assessment of Psoriasis and Psoriatic Arthritis; RCT, randomized clinical trial; ADA, adalimumab; ETN, etanercept; INF, infliximab; GOL, golimumab; CZP, certolizumab; UST, ustekinumab; SEC, secukinumab; IXE, ixekizumab; GUS, guselkumab; BRD, brodalumab; RIS, risankizumab; TIL, tildrakizumab; BRK, briakinumab; MTX, methotrexate; PLB, placebo*.

*①ACR20, ②ACR50, ③ACR70, ④PASI50, ⑤PASI75, ⑥PASI90, ⑦PASI100, ⑧dactylitis assessment, ⑨enthesitis assessment, ⑩nail assessment*.

*YES—the study was in NMA; NO—the study was not included in NMA*.

**Table 2:** Extension studies from RCT focusing on outcomes of GRAPPA domains.

**Study**	**Enrolled patients**						**NMA**
	**Author**	**Year**	***N***	**Time of outcome (weeks)**	**Drug**	**Dosage**	**Outcomes**
IMPACT 2 (58)	Kavanaugh	2007	200	52	IFX	5 mg/kg	①②③④
					PLB		⑤⑥⑧⑨
(59)	Menter	2008	1,212	52	ADA	40 mg	⑥⑦
					PLB		
REVEAL (60)	Gordon	2012	522	156	ADA	40 mg	PASI improvement
					PLB		
GO-REVEAL (61)	Kavanaugh	2012	405	52	GOL	50 mg	①②③⑤ ⑤⑧⑨⑩
					GOL	100 mg	
					PLB		
PHOENIX 1 (62)	Kimball	2012	766	156	UST	45 mg	④⑤⑥
					UST	90 mg	
					PLB		
GO-REVEAL (63)	Kavanaugh	2013	405	104	GOL	50 mg	①②③④
					GOL	100 mg	⑤⑧⑨⑩
					PLB		
PHOENIX 1 (64)	Kimball	2013	766	260	UST	45 mg	④⑤⑥
					UST	90 mg	
					PLB		
GO-REVEAL (65)	Kavanaugh	2014	405	268	GOL	50 mg	①②③④ ⑤⑧⑨⑩
					GOL	100 mg	
					PLB		
PSUMMIT 1	Kavanaugh	2014	927	52	UST	45 mg	Radiographic progression
PSUMMIT 2 (66)					UST	90 mg	
					PLB		
PSUMMIT 1 (67)	Kavanaugh	2015	615	100	UST	45 mg	①②③⑤
					UST	90 mg	⑥⑧⑨
					PLB		
PHOENIX 2 (68)	Langley	2015	1,212	260	UST	45 mg	⑤⑥
					UST	90 mg	
					PLB		
UNCOVER 3 (69)	Dennehy	2016	491	60	IXE	80 mg 2 w	⑩
					IXE	80 mg 4 w	
					ETN	50 mg	
					PLB		
UNCOVER 2 (70)	Gordon	2016	1,224	60	IXE	80 mg 2 w	⑤⑥⑦
					IXE	80 mg 4 w	
					ETN	50 mg	
					PLB		
UNCOVER 3 (70)	Gordon	2016	1,346	60	IXE	80 mg 2 w	⑤⑥⑦
					IXE	80 mg 4 w	
					ETN	50 mg	
					PLB		
(71)	van der Heijde	2016	606	52	SEC	10 mg/kg	Radiographic progression
					SEC	75 mg	
					SEC	150 mg	
PSTELLAR (72)	Blauvelt	2017	325	112	UST	q12 wk	⑤⑥⑦
					UST	q24 wk	
UNCOVER 3 (73)	Blauvelt	2017	1,346	108	IXE	80 mg 2 w	⑤⑥⑦
					IXE	80 mg 4 w	
					ETN	50 mg	
					PLB		
CLEAR (74)	Blauvelt	2017	676	52	SEC	300 mg	⑤⑥⑦
					UST	45/90 mg	
FUTURE 2 (75)	McInnes	2017	397	104	SEC	75 mg	①②③
					SEC	150 mg	⑥⑧⑨
					SEC	300 mg	
					PLB		
(76)	Mease	2017	422	54	TOF	5 mg	①②③
					TOF	10 mg	⑤⑧⑨
					ADA	40 mg	
					PLB		
LIBERATE (77)	Reich	2017	250	52	APR	30 mg	④⑤
					ETN	50 mg	⑥⑩
					PLB		
UNCOVER 3 (78)	van der Kerkhof	2017	809	60	IXE	80 mg 2 w	⑩
					IXE	80 mg 4 w	
					ETN	50 mg	
					PLB		
(79)	Griffiths	2018		100	GUS	100 mg	⑤⑥⑦
					ADA	40 mg	
					PLB		
UNCOVER 3 (80)	Leonardi	2018	1,346	156	IXE	80 mg 2 w	⑤⑥⑦
					IXE	80 mg 4 w	
					ETN	50 mg	
					PLB		
(81)	Ohtsuki	2018	191	52	GUS	50 mg	④⑤⑥
					GUS	100 mg	⑦⑩
					PLB		
LIBERATE (82)	Reich	2018	250	104	APR	30 mg	⑤⑩
					ETN	50 mg	
					PLB		
UNCOVER 2/3 (83)	Kemény	2019	2570	156	IXE	80 mg 2 w	⑤⑥⑦
					IXE	80 mg 4 w	
					ETN	50 mg	
					PLB		
IXORA-S (84)	Paul	2019	302	52	IXE	80 mg	⑤⑥⑦
					UST	45/90 mg	
SUSTaIMM (48)	Ohtsuki	2019	171	52	RIS	75 mg	⑤⑥⑦
					RIS	150 mg	
					PLB		

*N, number; NMA, network meta-analysis; GRAPPA, Group for Research and Assessment of Psoriasis and Psoriatic Arthritis; RCT, randomized clinical trial; ADA, adalimumab; ETN, etanercept; IFX, infliximab; GOL, golimumab; UST, ustekinumab; SEC, secukinumab; IXE, ixekizumab; GUS, guselkumab; RIS, risankizumab; APR, apremilast; TOF, tofacitinib; PLB, placebo*.

*①ACR20, ②ACR50, ③ACR70, ④PASI50, ⑤PASI75, ⑥PASI90, ⑦PASI100, ⑧dactylitis assessment, ⑨enthesitis assessment, ⑩nail assessment*.

### Peripheral Arthritis

The peripheral arthritis domain is predominantly assessed by instruments, such as ACR20, ACR50, and ACR70 criteria, which specify the improvement of 20, 50, or 70% in the number of tender and swollen joints, respectively, and a 20, 50, or 70% improvement in three of the following five criteria: patient global assessment, physician global assessment, functional ability measure (most often Health Assessment Questionnaire—HAQ), visual analog pain scale, and erythrocyte sedimentation rate or C-reactive protein (85). The main results of the ACR response in RCTs, at 24 weeks, are included in Table 3 (1, 11, 18, 24, 26, 28, 31, 32, 37, 38, 47, 51, 55). The head-to-head comparison of the ACR responses of SEC vs. ADA at week 52 in the EXCEED study is also listed but not included on the NMA (55).

**Table 3:** ACR improvements in patients with psoriatic arthritis—peripheral disease.

				**Improvement**	
			**ACR20**	**ACR50**	**ACR70**
**Study**	**Weeks**	**Treatment**	**n/total (%)**	**n/total (%)**	**n/total (%)**
Antoni 2005 IMPACT 2 (1)	24	PLB	16/100 (16.0)	4/100 (4.0)	2/100 (2.0)
	24	IFX	54/100 (54.0)	41/100 (41.0)	27/100 (27.0)
*p*-value			<0.001	<0.001	<0.001
Mease 2005 ADEPT (11)	24	PLB	15/162 (9.3)	6/162 (3.7)	1/162 (0.6)
	24	ADA	57/151 (37.7)	39/151 (25.8)	23/151 (15.2)
*p*-value			<0.001	<0.001	<0.001
Kavanaugh 2009 (18)	24	PLB	12/113 (10.6)	1/113 (0.9)	0/113 (0)
	24	GOL	75/146 (51.3)	39/146 (26.7)	25/146 (17.1)
*p*-value			<0.001	<0.001	<0.001
McInnes 2013 PSUMMIT 1 (24)	24	PLB	47/206 (22.8)	18/206 (8.7)	5/206 (2.4)
	24	UST	87/205 (42.4)	51/205 (24.8)	25/205 (12.2)
*p*-value			<0.0001	<0.0001	0.0001
Mease 2014 RAPID-PsA (26)	24	PLB	32/136 (23.5)	17/136 (12.5)	6/136 (4.4)
	24	CZP	88/138 (63.8)	60.1/138 (44.2)	39/138 (28.3)
*p*-value			<0.001	<0.001	<0.001
Ritchlin 2014 PSUMMIT 2 (28)	24	PLB	21/104 (20.2)	7/104 (6.7)	3/104 (2.9)
	24	UST	45/103 (43.7)	18/103 (17.4)	7/103 (6.8)
*p*-value			<0.001	<0.05	n.s.
Mease 2015 FUTURE 1 (32)	24	PLB	35/202 (17.3)	15/202 (7.4)	4/202 (2.0)
	24	SEC 150 mg	101/202 (50.0)	70/202 (34.7)	38/202 (18.8)
*p*-value			<0.001	<0.001	<0.001
McInnes 2015 FUTURE 2 (31)	24	PLB	15/98 (15.3)	7/98(7.1)	1/98 (1.0)
	24	SEC 300 mg*	54/100 (54.0)	35/100 (35.0)	20/100 (20.0)
	24	SEC 150 mg**	51/100 (51.0)	35/100 (35.0)	21/100 (21.0)
*p*-value			*,** <0.0001	*,** <0.0001	*0.0003; ** <0.0001
Mease 2017 SPIRIT P1 (37)	24	PLB	32/106 (30.2)	16/106 (15.1)	6/106 (5.7)
	24	IXE Q4W*	62/107 (57.9)	43/107 (40.2)	25/107 (23.4)
	24	ADA	58/101 (57.4)	39/101 (38.6)	26/101 (25.7)
*p*-value			* ≤ 0.001	* ≤ 0.001	* ≤ 0.001
Nash 2017 SPIRIT P2 (38)	24	PLB	23/118 (19.5)	6/118 (5.1)	0/118 (5.7)
	24	IXE Q4W	65/122 (53.3)	43/122 (35.2)	27/122 (22.1)
*p*-value			<0.0001	<0.0001	<0.0001
Mease 2018 FUTURE 5 (47)	24	PLB	78/332 (23.5)	29/332 (8.7)	13/332 (3.9)
	24	SEC 300 mg	141/222 (63.5)	97/222 (43.7)	56/222 (25.7)
		SEC 150 mg	117/220 (53.2)	86/220 (39.1)	53/220 (24.1)
*p*-value			<0.0001	<0.0001	<0.0001
Mease 2020 SPIRIT H2H (51)	24	ADA	204/283 (72.1)	132/283 (46.6)	73/283 (25.8)
	24	IXE	195/283 (68.9)	143/283 (50.5)	90/283 (31.8)
*p*-value			0.403	0.338	0.111
McInnes 2020 EXCEED (55)	52	SEC	285/426 (67)	209/426 (49)	141/426 (33)
	52	ADA	252/427 (43)	192/427 (45)	124/427 (29)
*p*-value			0.0239	0.2251	0.2950

ACR, American College of Rheumatology; n, number; ADA, adalimumab; IFX, infliximab; GOL, golimumab; CZP, certolizumab; UST, ustekinumab; SEC, secukinumab; IXE, ixekizumab; MTX, methotrexate; PLB, placebo *,**vs. placebo.

**Figure 3:**
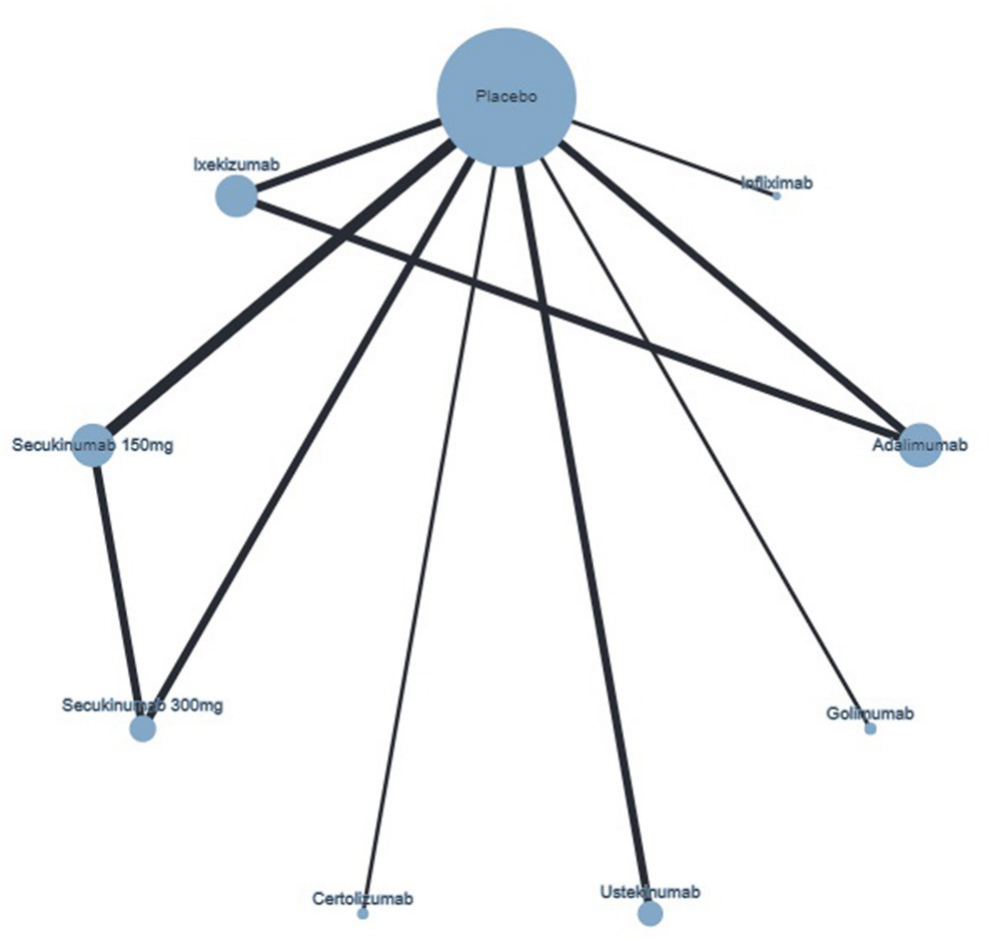
Network plot of ACR70 response showing direct comparisons, at week 24. The width of the edge is proportional to the number of studies, and the node size is proportional to the sample size.

An NMA was performed for the three outcomes (ACR20, ACR50, and ACR70). The included studies are identified in Table 1. A network plot for ACR70 is included in Figure 3, as an example of the network plots of these three NMAs.

The NMA results from the network of biologic therapies for the outcome ACR70 response are included in Table 4.

**Table 4:** NMA results from the network of biologic therapies in the outcome ACR70.

	ADA	CZP	GOL	IFX	IXE	PLB	SEC 150 mg	SEC 300 mg	UST
ADA		0.907 (0.274–3.002)	0.163 (0.009–3.013)	0.427 (0.081–2.264)	0.832 (0.607–1.139)	**7.745 (3.514–17.071)**	0.832 (0.323–2.144)	0.821 (0.314–2.148)	1.827 (0.594–5.625)
CZP	1.102 (0.333–3.646)		0.179 (0.009–3.425)	0.471 (0.084–2.633)	0.916 (0.276–3.041)	**8.535 (3.476–20.960)**	0.917 (0.324–2.590)	0.905 (0.316–2.592)	2.014 (0.605–6.705)
GOL	6.151 (0.332–114.000)	5.582 (0.292–106.708)		2.629 (0.110–62.633)	5.115 (0.276–94.907)	**47.641 (2.867–791.714)**	5.117 (0.294–89.211)	5.052 (0.288–88.517)	11.240 (0.605–208.847)
IFX	2.340 (0.442–12.395)	2.123 (0.380–11.870)	0.380 (0.016–9.064)		1.946 (0.366–10.329)	**18.123 (4.176–78.657)**	1.947 (0.410–9.241)	1.922 (0.401–9.207)	4.276 (0.804–22.753)
IXE	1.203 (0.878–1.648)	1.091 (0.329–3.622)	0.196 (0.011–3.628)	0.514 (0.097–2.729)		**9.315 (4.206–20.627)**	1.000 (0.387–2.588)	0.988 (0.376–2.593)	2.198 (0.712–6.788)
PLB	**0.129 (0.059–0.285)**	**0.117 (0.048–0.288)**	**0.021 (0.001–0.349)**	**0.055 (0.013–0.239)**	**0.107 (0.048–0.238)**		**0.107 (0.064–0.181)**	**0.106 (0.061–0.183)**	0.236 (0.106–0.525)
SEC 150 mg	1.202 (0.466–3.098)	1.091 (0.386–3.082)	0.195 (0.011–3.407)	0.514 (0.108–2.439)	1.000 (0.386–2.586)	**9.310 (5.529–15.679)**		0.987 (0.688–1.418)	2.197 (0.846–5.706)
SEC 300 mg	1.218 (0.466–3.184)	1.105 (0.386–3.164)	0.198 (0.011–3.468)	0.520 (0.109–2.493)	1.012 (0.386–2.658)	**9.430 (5.455–16.302)**	1.013 (0.705–1.454)		2.225 (0.844–5.864)
UST	0.547 (0.178–1.685)	0.497 (0.149–1.653)	0.089 (0.005–1.653)	0.234 (0.044–1.244)	0.455 (0.147–1.405)	4.238 (1.905–9.431)	0.455 (0.175–1.182)	0.449 (0.171–1.185)	

*OR and CrI are presented. Comparisons with high confidence rating based on CINeMA evaluation are identified in bold and OR higher than 1 favor the intervention specified in the row*.

*NMA, network meta-analysis; CINeMA, Confidence in Network Meta-Analysis; OR, odds ratio; CrI, credible interval; ADA, adalimumab; IFX, infliximab; GOL, golimumab; CZP, certolizumab; UST, ustekinumab; SEC, secukinumab; IXE, ixekizumab; PLB, placebo*.

### Axial Disease

Data including biologic therapies for axial disease, in the context of PsD, are scarce, possibly because there is no validated instrument to assess this domain. Nowadays, the only trial addressing specifically PsD patients with the axial disease is still ongoing and this data is not yet published. This trial—MAXIMIZE—evaluates the efficacy and safety of SEC 300 or 150 mg in managing axial manifestations in patients with PsA, who have failed to respond to at least 2 non-steroidal anti-inflammatory drugs (NSAIDs) over 4 weeks, according to Assessment of Spondyloarthritis International Society (ASAS) recommendations for the treatment of axial spondyloarthritis (ClinicalTrials.gov NCT02721966) (86).

### Enthesitis

There are at least 6 indices to evaluate enthesitis outcomes (4-point enthesitis measure, Leeds Enthesis Index (LEI), Maastricht Ankylosing Spondylitis Enthesitis Score (MASES), Spondyloarthritis Research Consortium of Canada (SPARCC) Enthesitis Index, 12-point Berlin Index, and the 17-point University of California, San Francisco (UCSF) Index) with no consensus on which is the most adequate (85). Moreover, some studies, instead of using a score, only discriminate the percentage of patients with complete enthesitis resolution. Since different instruments were used in different studies, it is impossible to compare results across studies. As such, we were not able to perform an NMA regarding this domain. A summary of the results of the different studies is included in Table 5 (1, 13, 18, 22, 24, 26, 28, 31, 32, 37, 38, 47, 50, 51, 55, 61, 65).

**Table 5:** Enthesitis assessment in patients with psoriatic arthritis.

**Study**	**Author**	**Year**	**Drug**	**Outcome**	**Time of outcome (weeks)**	**Result**
IMPACT 2 (1)	Antoni	2005	IFX vs. PLB	Patients with enthesitis	14	22% vs. 34% (*p =* 0.016)
					24	20% vs. 37% (*p =* 0.002)
(13)	Genovese	2007	ADA vs. PLB	Reduction of enthesitis	12	−0.5 vs. −0.2 (*p >* 0.05)
GO-REVEAL (18)	Kavanaugh	2009	GOL 50 mg vs. PLB	Patients with enthesitis	14	55% vs. 71% (*p =* 0.008)
			GOL 100 mg vs. PLB			61% vs. 71% (*p =* 0.10)
			GOL 50 mg vs. PLB		24	49% vs. 69% (*p =* 0.002)
			GOL 100 mg vs. PLB			50% vs. 69% (*p =* 0.003)
GO-REVEAL (61)	Kavanaugh	2012	GOL 50 mg vs. PLB	Modified MASES index (change from baseline)	Week 52	56.3 ± 62.4 vs. 39.1 ± 76.1
			GOL100 mg vs. PLB			51.9 ± 64.2 vs. 39.1 ± 76.1
RESPOND (22)	Baranauskaite	2012	IFX+MTX vs. MTX	Reduction of enthesitis	16	2 vs. 1 (*p =* 0.082)
PSUMMIT 1 (24)	McInnes	2013	UST vs. PLB	Patients with enthesitis	24	64.6% vs. 81% (*p =* 0.006)
GO-REVEAL (64)	Kavanaugh	2014	GOL 50 mg vs. PLB	Modified MASES index	Week 256	1.9 ± 3.3 vs. 2.4 ± 4.0
			GOL100 mg vs. PLB			2.0 ± 3.4 vs. 2.4 ± 4.0
PSUMMIT 2 (28)	Ritchlin	2014	UST 45 mg vs. PLB	MASES	24	−33,33% vs. 0% (*p >* 0.05)
			UST 90 mg vs. PLB			−48.33% vs. 0% (p <0.01)
			UST 45 mg vs. PLB		52	−36.67% vs. −33.33% (*p >* 0.05)
			UST 90 mg vs. PLB			−60% vs. −33.33% (*p >* 0.05)
RAPID-PsA (26)	Mease	2014	CZP 200 mg Q2W vs. PLB	LEI	24	−2.0 vs. −1.1 (p <0.001)
			CZP 400 mg Q4W vs. PLB	LEI		−1.8 vs. −1.1 (*p =* 0.003)
(32)	Mease	2015	SEC (pooled data) vs. PLB	Resolution of enthesitis	24	47.5% vs. 12.8% (p <0.05)
FUTURE 2 (31)	McInnes	2015	SEC (pooled data) vs. PLB	Resolution of enthesitis	24	22% vs. 40% (*p =* 0.919)
SPIRIT-P1 (37)	Mease	2017	IXE Q2W vs. PLB vs. ADA	LEI (responders)	Week 12	47.4 vs. 28.1 vs. 35.2 (p <0.05)*
			IXE Q4W vs. PLB vs. ADA			27.9 vs. 28.1 vs. 35.2
			IXE Q2W vs. PLB vs. ADA		Week 24	38.6 vs. 19.3 vs. 33.3 (*p ≤* 0.025)*
			IXE Q4W vs. PLB vs. ADA			42.6 vs. 19.3 vs. 35.2 (*p ≤* 0.01)*
SPITIT-P2 (38)	Nash	2017	IXE Q2W vs. PLB	LEI (proportion of patients with a response)	Week 24	31% vs. 22% (*p =* 0.27)
			IXE Q4W vs. PLB			35% vs. 22% (*p =* 0.08)
FUTURE 5 (47)	Mease	2018	SEC 150 mg vs. PLB	Resolution of enthesitis	Week 16	54.6% vs. 35.4% (*p < * 0.05)
			SEC 300 mg vs. PLB			55.7% vs. 35.4% (*p <* 0.05)
ECLIPSA (50)	Araujo	2019	UST vs. TNFi	SPARCC = 0	Week 12	74% vs. 42% (*p =* 0.018)
				MASES = 0		82% vs. 50% (*p =* 0.032)
				LEI = 0		78% vs. 50% (*p =* 0.005)
SPIRIT H2H (51)	Mease	2020	IXE vs. ADA	SPARCC = 0	Week 24	45.0% vs. 56.6% (*p =* 0.019)
				LEI = 0		55.1% vs. 59.7% (*p =* 0.432)
EXCEED (55)	McInnes	2020	SEC vs. ADA	resolution of enthesitis	Week 52	53% vs. 50% (*p =* 0.5117)

ADA, adalimumab; IFX, infliximab; GOL, golimumab; CZP, certolizumab; UST, ustekinumab; SEC, secukinumab; IXE, ixekizumab; BRD, brodalumab; MTX, methotrexate; PLB, placebo; MASES, Maastricht Ankylosing Spondylitis Enthesitis Score; LEI, Leeds Enthesitis Index; SPARCC, Spondyloarthritis Research Consortium of Canada Enthesitis Index; TNFi, tumor necrosis factor inhibitor; ^*^IXE vs. placebo.

### Dactylitis

As enthesitis, dactylitis is also evaluated through different approaches. It can be assessed by counting dactylitis digits—a simple counting and scoring method or Leeds Dactylitis Index (LDI) (85). Moreover, there is also no consensus regarding the better method to assess dactylitis, and therefore it was not possible to perform an NMA due to the heterogeneity found in the different RCTs (85). Also, some studies only evaluate the percentage of patients with complete resolution of dactylitis. A summary of the results of the different studies is included in Table 6 (1, 18, 24, 26, 28, 31, 32, 37, 38, 47, 51, 55, 61, 65).

**Table 6:** Dactylitis assessment in patients with psoriatic arthritis.

**Study**	**Author**	**Year**	**Intervention**	**Outcome**	**Time of outcome**	**Result**
IMPACT 2 (1)	Antoni	2005	IFX vs. placebo	At least 1 dactylitis digit	Week 14	22% vs. 34% (*p =* 0.025)
					Week 24	12% vs. 34% (p <0.001)
GO-REVEAL (18)	Kavanaugh	2009	GOL 50 mg vs. placebo	Patients with dactylitis	Week 14	22% vs. 26% (*p =* 0.46)
			GOL 100 mg vs. placebo			17% vs. 26% (*p =* 0.10)
			GOL 50 mg vs. placebo		Week 24	16% vs. 22% (*p =* 0.21)
			GOL 100 mg vs. placebo			14% vs. 22% (*p =* 0.09)
GO-REVEAL (61)	Kavanaugh	2012	GOL 50 mg vs. placebo	Dactylitis score change from baseline	Week 52	−4.20 ± 4.81 vs. −1.68 ± 2.79
			GOL 100 mg vs. placebo			−4.55 ± 6.60 vs. −1.68 ± 2.79
PSUMMIT 1 (24)	McInnes	2013	UST vs. placebo	Patients with dactylitis	Week 24	56.2% vs. 76.1% (*p =* 0.0013)
PSUMMIT 2 (28)	Ritchlin	2014	UST 45 mg vs. placebo	Percent change in dactylitis score	Week 24	0.0 vs. 0.0
			UST 90 mg vs. placebo			−64.58 vs. 0.0
			UST 45 mg vs. placebo		Week 52	−95.00 vs. −100
RAPID-PsA (26)	Mease	2014	CZP 200 mg Q2W vs. placebo	LDI change from baseline	Week 24	−40.7 vs. −22.0 (*p =* 0.002)
			CZP 400 mg Q4W vs. placebo			−53.5 vs. −22.0 (p <0.001)
GO-REVEAL (65)	Kavanaugh	2014	GOL 50 mg vs. placebo	Dactylitis score	Week 260	6.3 ± 6.1 vs. 3.1 ± 2.1
			GOL 100 mg vs. placebo			5.4 ± 6.7 vs. 3.1 ± 2.1
			UST 90 mg vs. placebo			−90.91 vs. −100
(30)	Mease	2015	SEC (pooled data) vs. placebo	Resolution of dactylitis	Week 24	52.4% vs. 15.5 (p <0.05)
FUTURE 2 (31)	McInnes	2015	SEC (pooled data) vs. placebo	Resolution of dactylitis	Week 24	47% vs. 15% (*p =* 0.919)
SPIRIT-P1 (37)	Mease	2017	IXE Q2W vs. placebo vs. ADA	LDI-B (change from baseline)	Week 12	−63.9 (10.6) vs. −36.3 (10.3) vs. −62.1 (11.9) (*p ≤* 0.05)*
			IXE Q4W vs. placebo vs. ADA			−72.8 (8.8) vs. −36.3 (10.3) −62.1 (11.9) (*p ≤* 0.001)*
			IXE Q2W vs. placebo vs. ADA		Week 24	−66.1 (9.8) vs. −33.7 (9.7) vs. −76.0 (10.9) (*p ≤* 0.01)*
			IXE Q4W vs. placebo vs. ADA			−75.4 (8.1) vs. −33.7 (9.7) −76.0 (10.9) (*p ≤* 0.001)*
SPIRIT-P2 (38)	Nash	2017	IXE Q2W vs. placebo	LDI-B (change from baseline)	Week 24	−32.1 (6.7) vs. −36.2 (8.4) *p =* 0.65
			IXE Q4W vs. placebo			−34.7 (6.7) VS. −36.2 (8.4) *p =* 0.85
FUTURE 5 (47)	Mease	2018	SEC 150 mg vs. placebo	Resolution of dactylitis	Week 16	57.5% vs. 32.3% (*p <* 0.05)
			SEC 300 mg vs. placebo			65.9% vs. 32.3% (*p <* 0.05)
SPIRIT H2H (51)	Mease	2020	IXE vs. ADA	LDI-B = 0	Week 24	88.1 vs. 93.1 (*p =* 0.658)
EXCEED (55)	McInnes	2020	SEC vs. ADA	Resolution of dactylitis	Week 52	75% vs. 70% (*p =* 0.3560)

ADA, adalimumab; IFX, infliximab; GOL, golimumab; CZP, certolizumab; UST, ustekinumab; SEC, secukinumab; IXE, ixekizumab; BRD, brodalumab; PLB, placebo; LDI, Leeds Dactylitis Index; *IXE vs. placebo.

### Skin

Psoriasis severity was evaluated by the most used tool in dermatology trials—PASI. PASI combines the assessment of the severity of psoriasis lesions (average redness, thickness, and scaliness of the lesions) and the area affected into a single score (87). PASI is commonly reported as the percentage of improvement from baseline, PASI75, PASI90, and PASI100, meaning 75, 90, and 100% of improvement, respectively.

The results of the systematic review including RCTs reporting PASI in patients with PsD, at weeks 10–16 of treatment, are included in Table 7 (1, 11, 12, 14–16, 18–22, 25, 26, 29, 30, 33, 35–37, 39–41, 43–48, 51–53, 55–57, 59, 70).

**Table 7:** PASI Improvements in patients with psoriasis skin.

				**Improvement**	
			**PASI75**	**PASI90**	**PASI100**
**Study**	**Weeks**	**Drug**	**n/total (%)**	**n/total (%)**	**n/total (%)**
Antoni 2005	14	PLB	1/87 (1.0)	0/87 (0.0)	–
IMPACT 2 (1)	14	IFX	55/87 (64.0)	34/87 (41.0)	–
*p*-value			<0.001	<0.001	
Mease 2005	12	PLB	4/69 (5.8)	0/69 (0.0)	–
ADEPT (11)	12	ADA	49/69 (71.0)	30/69 (43.5)	–
*p*-value			<0.001	<0.001	
Reich 2005	10	PLB	2/77 (3.0)	1/77 (1.0)	–
(12)	10	IFX	242/301 (80.0)	172/301 (57.0)	–
*p*-value			<0.0001	<0.0001	
Tyring 2007	12	PLB	5/292 (1.7)	1/292 (0.3)	–
(13)	12	ETA	47/305 (15.4)	21/305 (6.9)	–
*p*-value			<0.001	<0.001	
Leonardi 2008	12	PLB	5/255 (2.0)	5/255 (2.0)	0/255 (0.0)
PHOENIX 1 (15)	12	UST 45 mg	171/255 (67.0)	106/255 (41.6)	32/255 (12.5)
*p*-value			<0.0001	<0.0001	<0.0001
Menter 2008	12	PLB	20/398 (5.0)	8/398 (2.0)	4/398 (1.0)
(59)	12	ADA	554/814 (68.1)	301/814 (37.0)	114/814 (14.0)
*p*-value			<0.001	<0.001	<0.001
Papp 2008	12	PLB	15/410 (3.7)	3/410 (0.7)	0/410 (0.0)
PHOENIX 2 (16)	12	UST 45 mg	273/409 (66.5)	173/409 (42.3)	74/409 (18.1)
*p*-value			<0.0001	<0.0001	<0.0001
Kavanaugh 2009	14	PLB	2/79 (2.5)	0/73 (0.0)	–
(18)	14	GOL 50 mg	44/109 (40.3)	22/106 (20.8)	–
*p*-value			<0.001	<0.001	
Barker 2011	16	MTX	90/216 (41.7)	41/216 (19.0)	–
RESTORE1 (19)	16	IFX	508/656 (77.4)	356/656 (54.2)	–
*p*-value			n.s.	<0.001	
Gottlieb 2011	12	PLB	5/68 (7.4)	1/68 (1.5)	0/68 (0.0)
(20)	12	BRK*	112/138 (81.0)	83/138 (60.0)	39/138 (28.3)
	12	ETA	78/141 (55.0)	18/141 (12.7)	5/141 (3.6)
*p*-value			<0.001	<0.001	<0.001
Strober 2011	12	PLB	5/72 (6.9)	3/72 (4.2)	0/72 (0.0)
(20)	12	BRK*	111/139 (80.0)	83/139 (60)	30/139 (21.9)
	12	ETA	40/139 (28.8)	18/139 (13.0)	5/139 (3.6)
*p*-value			<0.001	<0.001	<0.001
Baranauskaite 2012	16	MTX	19/35 (54.3%)	–	–
RESPOND (22)	16	MTX+IFX	33/34 (97.1%)	–	–
*p*-value			<0.0001	–	–
Langley 2014	12	PLB	11/246 (4.5)	3/246 (1.2)	2/246 (0.8)
ERASURE (25)	12	SEC 300 mg	200/245 (81.6)	145/245 (59.2)	70/245 (26.6)
*p*-value			<0.001	<0.001	<0.001
Langley 2014	12	PLB	16/324 (4.9)	5/324 (1.5)	0/324 (0.0)
FIXTURE (25)	12	SEC 300 mg*	249/323 (77.0)	175/323 (54.1)	78/323 (24.1)
	12	ETA	142/323 (44.0)	67/323 (20.7)	14/323 (4.3)
*p*-value			<0.001	<0.001	<0.001
Mease 2014	12	PLB	12/86 (13.9)	4/86 (4.7)	–
RAPID-PsA (26)	12	CZP 200 mg	42/90 (46.7)	20/90 (22.2)	–
*p*-value			<0.001	<0.001	
Griffiths 2015 UNCOVER 2 (29)	12	PLB	4/168 (2.4)	1/168 (0.6)	1/168 (0.6)
	12	IXE Q4W*	269/347 (77.5)	267/347 (76.9)	107/347 (30.8)
	12	ETA	149/358 (41.6)	67/358 (18.7)	19/358 (5.3)
*p*-value			<0.0001	<0.0001	<0.0001
Griffiths 2015 UNCOVER 3 (29)	12	PLB	14/193 (7.2)	6/193 (3.1)	0/193 (0.0)
	12	IXE*	325/386 (84.2)	352/386 (91.2)	135/386 (35.0)
	12	ETA	201/382 (52.6)	98/382 (25.6)	19/358 (5.3)
*p*-value			<0.0001	<0.0001	<0.0001
Lebwohl 2015	12	PLB	25/309 (8.1)	12/309 (3.9)	2/309 (0.6)
AMAGINE 2 (30)	12	UST	210/300 (70.0)	141/300 (47.0)	65/300 (21.7)
	12	BRD 210 mg*	528/612 (86.3)	428/612 (69.9)	272/612 (44.4)
*p*-value			<0.001	<0.001	<0.001
Lebwohl 2015	12	PLB	19/315 (6.0)	6/315 (1.9)	1/315 (0.3)
AMAGINE 3 (30)	12	UST	217/313 (69.3)	141/313 (45.0)	58/313 (18.5)
	12	BRD*	531/624 (85.1)	430/624 (68.9)	229/624 (36.7)
*p*-value			<0.001	<0.001	<0.001
Thaci 2015	12	SEC	311/334 (93.1)	264/334 (79.0)	148/334 (44.3)
CLEAR (33)	12	UST	277/334 (82.9)	277/334 (82.9)	130/334 (38.9)
*p*-value			<0.0001	<0.0001	=0.003
Gordon 2016	12	PLB	17/431 (3.9)	7/431 (1.7)	0/431 (0.0)
UNCOVER 1 (70)	12	IXE Q4W	357/432 (82.6)	279/432 (64.6)	145/432 (33.6)
*p*-value			<0.001	<0.001	<0.001
Papp 2016	12	PLB	6/220 (2.7)	2/220 (0.9)	1/220 (0.5)
(35)	12	BRD	185/222 (83.3)	156/220 (70.9)	93/222 (41.9)
*p*-value			<0.001	<0.001	<0.001
Blauvelt 2017	16	PLB	10/174 (5.7)	5/174 (2.9)	1/174(0.6)
VOYAGE 1	16	GUS*	300/329 (91.2)	241/329 (73, 3)	123/329 (37.4)
(36)	16	ADA	244/334 (73.1)	166/334 (49.7)	57/334 (17.4)
*p*-value			<0.001	<0.001	<0.001
Mease 2017	12	PLB	5/67 (7.5)	1/67 (1.5)	1/67 (1.5)
SPIRIT 1	12	IXE Q4W*	55/73 (75.3)	38/73 (52.0)	23/73 (31.5)
(37)	12	ADA	23/68 (33.8)	15/68 (22.1)	10/68 (14.7)
*p*-value			≤ 0.01	≤ 0.01	≤ 0.01
Reich 2017	12	PLB	9/154 (5.8)	4/154 (3.0)	2/154 (1.3)
reSURFACE 1 (39)	12	TIL 100 mg	197/309 (63.8)	107/309 (35.0)	43/309 (13.9)
*p*-value			<0.0001	<0.0001	<0.0001
Reich 2017	12	PLB	9/156 (5.8)	2/156 (1.3)	0/156 (0.0)
reSURFACE 2 (39)	12	TIL 100 mg*	188/307 (61.2)	119/307 (38.8)	38/307 (12.4)
	12	ETA	151/313 (48.2)	67/313 (21.4)	15/313 (4.8)
*p*-value			0.0001	0.0001	0.0001
Reich 2017	12	IXE	120/136 (88.2)	99/136 (72.8)	49/136 (36.0)
IXORA-S (40)	12	UST	114/166 (68.7)	70/166 (42, 2)	24/166 (14.5)
*p*-value			0.001	0.001	0.001
Bagel 2018	16	SEC	504/550 (91.7)	421/550 (76.6)	249/550 (45.3)
CLARITY (41)	16	UST	440/552 (79.8)	299/552 (54.1)	147/552 (26.7)
*p*-value			<0.0001	<0.0001	<0.0001
Gordon 2018	12	PLB	10/102 (9.8)	2/102 (2.0)	0/102 (0.0)
UltiMMa 1 (43)	12	UST*	70/100 (70)	42/100 (42.0)	12/100 (12.0)
	12	RIS*	264/304 (86.8)	229/304 (75.3)	109/304 (35.9)
*p*-value			<0.0001	<0.0001	<0.0001
Gordon 2018	12	PLB	8/98 (8.1)	2/98 (2.0)	2/98 (2.0)
UltiMMa 2 (43)	12	UST	69/99 (69.7)	47/99 (47.5)	24/99 (24.2)
	12	RIS	261/294 (88.8)	220/294 (74.9)	149/294 (50.7)
*p*-value			<0.0001	<0.0001	<0.0001
Gottlieb 2018	16	PLB	3/51 (6.5)	0/51 (0.0)	0/51 (0.0)
CIMPASI 1 (44)	16	CZP 200 mg	63/95 (66.3)	34/95 (35.8)	13/95 (13.7)
*p*-value			<0.0001	<0.0001	<0.0001
Gottlieb 2018	16	PLB	6/49 (11.6)	2/49 (2.2)	1/49 (1.8)
CIMPASI 2 (44)	16	CZP 200 mg	74/92 (81.4)	48/91 (52.6)	14/91 (15.4)
*p*-value			<0.0001	<0.0001	<0.0001
Lebwohl 2018	16	PLB	3/57 (5.3)	5/57 (0.0)	–
CIMPACT (45)	16	CZP 200 mg	113/165 (68.5)	66/165 (40.0)	–
*p*-value			<0.0001	<0.0001	
Reich 2018	16	PLB	3/65 (4.6)	1/65 (1.5)	0/65 (0.0)
TRANSFIGURE (46)	16	SEC 300 mg	56/66 (84.8)	48/66 (72.7)	22/66 (33.3)
*p*-value			<0.001	<0.001	
Mease 2018	16	PLB	40/332 (12.3)	31/332 (9.3)	–
FUTURE 5 (47)	16	SEC 150 mg	132/220 (60.0)	81/220 (36.8)	–
	16	SEC 300 mg	155/222 (70.0)	119/222 (53.6)	–
*p*-value			<0.05	<0.05	
Reich 2019	16	RIS 150 mg	237/301 (91)	218/301 (72)	120/301 (40)
IMMvent (52)	16	ADA	218/304 (72)	144/304 (47)	70/304 (23)
*p*-value			<0.0001	<0.0001	<0.0001
Reich 2019	12	GUS	477/534 (89)	369/534 (69)	311/534 (58)
ECLIPSE (53)	12	SEC	471/514 (92)	391/514 (76)	249/514 (48)
*p*-value			NA	NA	NA
Ohtsuki 2019 SustaIMM (48)	16	RIS 75 mg*	52/58 (89.8)	–	13/58 (22.4)
	16	RIS 150 mg*	52/55 (94.5)	–	18/55 (32.7)
	16	PLB	5/58 (8.6)	–	0/0
*p*-value			<0.001		<0.001
Mease 2020	16	ADA	195/238 (68.9)	158/283 (55.8)	132/283 (46.6)
SPIRIT H2H (51)	16	IXE	227/283 (80.2)	203/283 (71.7)	170/283 (60.1)
*p*-value			*p =* 0.002	<0.001	<0.001
McInnes 2020	52	SEC	170/215 (79)	140/215 (54)	99/215 (46)
EXCEED (55)	52	ADA	123/202 (61)	87/202 (43)	61/202 (30)
*p*-value			0.0002	<0.0001	0.0007
Ferris 2020	16	GUS	55/62 (88.7)	47/62 (75.8)	31/62 (50.0)
ORION (56)	16	PLB	0/16 (0)	0/16 (0)	0/16 (0)
*p*-value			<0.001	<0.001	<0.001
Warren 2020	16	RIS	92/164 (56.1)	74/164 (45.1)	44/164 (26.9)
IMMerge (57)	16	SEC	80/163 (49.1)	66/163 (40.5)	34/163 (20.9)

PASI, Psoriasis Area Severity Index; ADA, adalimumab; ETN, etanercept; IFX, infliximab; GOL, golimumab; CZP, certolizumab; UST, ustekinumab; SEC, secukinumab; IXE, ixekizumab; GUS, guselkumab; BRD, brodalumab; RIS, risankizumab; TIL, tildrakizumab; BRK, briakinumab; MTX, methotrexate; PLB, placebo; *vs. placebo.

**Figure 4:**
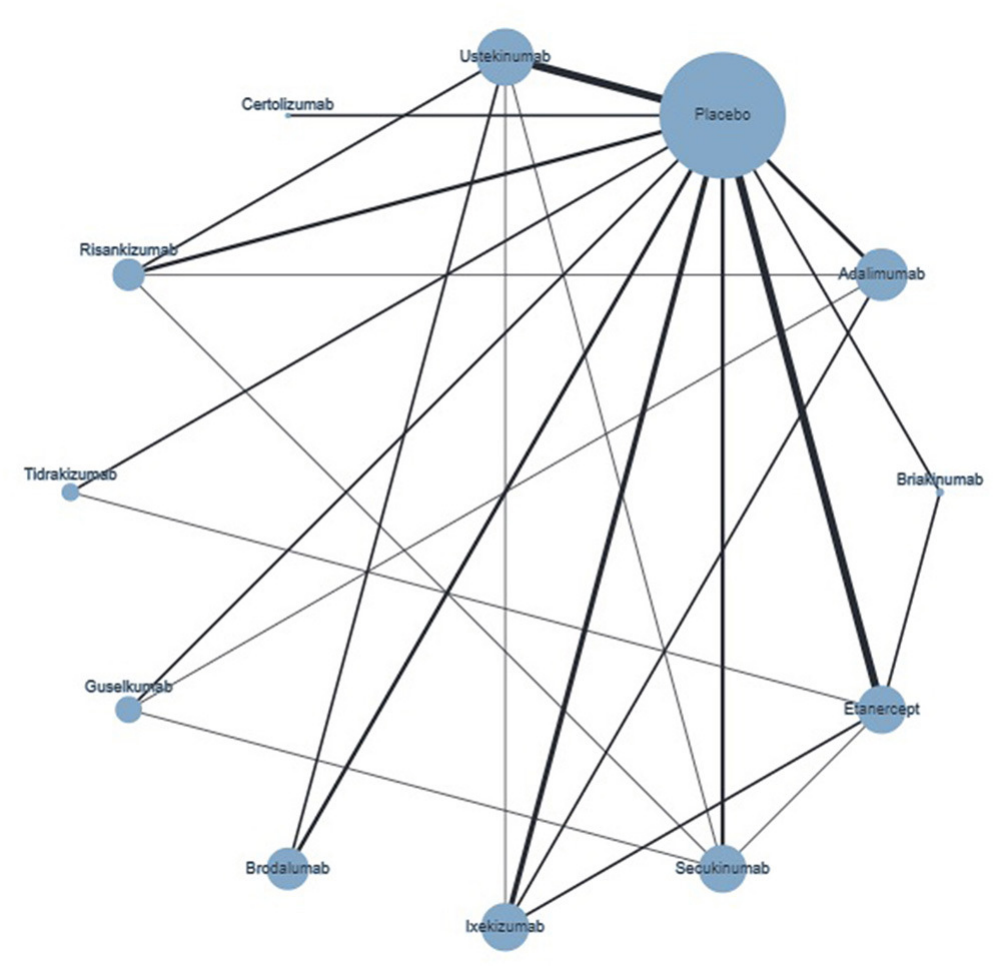
Network plot of PASI100 showing direct comparisons, at weeks 10–16. The width of the edge is proportional to the number of studies, and the node size is proportional to the sample size.

An NMA was performed for the three outcomes: PASI75, PASI90, and PASI100. The included studies are identified in Table 1. A network plot for PASI100 is included in Figure 4, as an example of the network plots of these three NMAs.

### Nails

As described for enthesitis and dactylitis, the assessment of nail psoriasis is not consensual at this time, with Nail Psoriasis Severity Index (NAPSI) and modified NAPSI being the most commonly used indices. Due to the low number of studies evaluating nail psoriasis and inconsistent use of these indices, we were not able to perform an NMA (85). A summary of the results of the different studies is included in **Table 9** (12, 18, 27, 32, 34, 36–38, 42, 46, 51, 61, 65, 69, 77, 78, 81, 88, 89).

## Discussion

The use of biologic therapies in the treatment of PsD is recommended across the six domains of the disease (2). A complete, effective, and safe treatment for all the manifestations of PsD is the main goal in the management of this condition. However, the heterogeneity of the manifestations challenges the achievement of this goal.

Recent advances in the knowledge of the pathophysiology of the disease led to the extensive study and approval of different mechanisms of action, including TNFi such as IFX, ETN, GOL, CZP, and ADA; IL-17i such as SEC, IXE, and BRD; and IL-12 and/or IL23i such as UST, GUS, RIS, and TIL. Nevertheless, direct comparisons between them are scarce and therefore NMA is the preferred method to indirectly compare drugs, aiming to help clinicians in the choice of the best treatment.

The report of the outcomes of each GRAPPA domain is not standardized (Tables 5, 6, **9**) except for the peripheral arthritis and skin domains, which use mainly ACR and PASI responses, respectively (Tables 3, 7). Thus, we were only able to perform NMAs based on ACR and PASI responses, evaluated at weeks 24 or 10–16, respectively. Although we also performed NMAs for ACR20, ACR50, PASI75, and PASI90, based on the current expectations on the efficacy of new biologic treatments and on the confidence in the results, we decided to present the efficacy of the different biologic therapy using ACR70 (Table 4) and PASI100 (Table 8), the most challenging outcomes. The confidence rating on direct and indirect estimates was calculated using CINeMA to improve the transparency and limit the subjectivity of the process (90–92). Comparisons with a high confidence rating, based on the CINeMA evaluation (91), are represented in bold. The level of confidence of the other comparisons is either low or very low, and consequently, the surface under the cumulative rating (SUCRA) will result in misleading inferences (90, 93). Thus, a SUCRA was not done and, therefore, it was impossible to rank the available biologic treatments.

**Table 8:** NMA results from the network of biologic therapies in the outcome PASI100.

	ADA	BRK	BRD	CZP	ETN	GUS	IXE	PLB	RIS	SEC	TIL	UST
ADA		0.511 (0.247–1.057)	**0.412 (0.279–0.610)**	3.005 (0.531–16.998)	**4.078 (2.825–5.887)**	**0.518 (0.363–0.738)**	**0.514 (0.393–0.671)**	**32.891 (20.602–52.505)**	**0.426 (0.320–0.567)**	0.767 (0.556–1.058)	1.590 (0.830–3.047)	1.152 (0.831–1.597)
BRK	1.956 (0.946–4.046)		0.807 (0.374–1.740)	5.877 (0.929–37.192)	**7.977 (4.211–15.111)**	1.013 (0.476–2.155)	1.005 (0.501–2.017)	**64.335 (29.280–141.359)**	0.833 (0.395–1.758)	1.500 (0.722–3.116)	**3.110 (1.332–7.263)**	**2.253 (1.076–4.717)**
BRD	**2.424 (1.640–3.548)**	1.239 (0.575–2.673)		**7.285 (1.276–41.571)**	**9.887 (6.322–15.462)**	1.256 (0.847–1.862)	1.245 (0.837–1.854)	**79.742 (48.415–131.341)**	1.033 (0.718–1.486)	**1.859 (1.329–2.601)**	**3.855 (1.925–7.718)**	**2.793 (2.230–3.498)**
CZP	0.333 (0.059–1.883)	0.170 (0.027–1.077)	**0.137 (0.024–0.783)**		1.357 (0.237–7.759)	0.172 (0.030–0.984)	0.171 (0.030–0.969)	**10.946 (2.063–58.067)**	**0.142 (0.025–0.805)**	0.255 (0.045–1.444)	0.529 (0.087–3.225)	0.383 (0.068–2.168)
ETN	**0.245 (0.170–0.354)**	**0.125 (0.066–0.237)**	**0.101 (0.065–0.158)**	0.737 (0.129–4.212)		**0.127 (0.083–0.193)**	**0.126 (0.094–0.169)**	**8.066 (4.866–13.368)**	**0.104 (0.070–0.157)**	**0.188 (0.129–0.274)**	**0.390 (0.222–0.686)**	**0.282 (0.191–0.418)**
GUS	**1.931 (1.355–2.752)**	0.987 (0.464–2.100)	0.796 (0.537–1.181)	5.801 (1.016–33.129)	**7.873 (5.169–11.994)**		0.992 (0.678–1.452)	**63.510 (38.479–104.815)**	**0.823 (0.576–1.175)**	**1.481 (1.177–1.862)**	**3.070 (1.554–6.065)**	**2.224 (1.602–3.087)**
IXE	**1.947 (1.490–2.544)**	0.995 (0.496–1.998)	0.803 (0.539–1.195)	5.849 (1.032–33.159)	**7.938 (5.908–10.667)**	1.008 (0.689–1.476)		**64.027 (39.805–102.997)**	0.829 (0.589–1.168)	**1.493 (1.063–2.095)**	**3.095 (1.664–5.758)**	**2.243 (1.605–3.134)**
PLB	**0.030 (0.019–0.049)**	**0.016 (0.007–0.034)**	**0.013 (0.008–0.021)**	**0.091 (0.017–0.485)**	**0.124 (0.075–0.206)**	**0.016 (0.010–0.026)**	**0.016 (0.010–0.025)**		**0.013 (0.008–0.021)**	**0.023 (0.015–0.037)**	**0.048 (0.024–0.097)**	**0.035 (0.022–0.056)**
RIS	**2.347 (1.763–3.125)**	1.200 (0.569–2.531)	0.968 (0.673–1.393)	**7.052 (1.242–40.037)**	**9.571 (6.376–14.368)**	**1.216 (0.851–1.736)**	1.206 (0.856–1.698)	**77.192 (47.727–124.861)**		**1.800 (1.330–2.436)**	**3.732 (1.908–7.299)**	**2.704 (2.022–3.616)**
SEC	1.304 (0.945–1.800)	0.667 (0.321–1.385)	**0.538 (0.385–0.753)**	3.919 (0.693–22.174)	**5.319 (3.650–7.749)**	**0.675 (0.537–0.850)**	**0.670 (0.477–0.940)**	**42.897 (26.848–68.539)**	**0.556 (0.411–0.752)**		**2.074 (1.077–3.992)**	1.502 (1.165–1.938)
TIL	0.629 (0.328–1.205)	**0.322 (0.138–0.751)**	**0.259 (0.130–0.519)**	1.890 (0.310–11.518)	**2.565 (1.458–4.511)**	**0.326 (0.165–0.643)**	**0.323 (0.174–0.601)**	**20.687 (10.326–41.438)**	**0.268 (0.137–0.524)**	**0.482 (0.251–0.928)**		0.724 (0.374–1.405)
UST	0.868 (0.626–1.203)	**0.444 (0.212–0.929)**	**0.358 (0.286–0.448)**	2.608 (0.461–14.746)	**3.540 (2.390–5.242)**	**0.450 (0.324–0.624)**	**0.446 (0.319–0.623)**	**28.551 (17.930–45.468)**	**0.370 (0.277–0.495)**	0.666 (0.516–0.858)	1.380 (0.712–2.676)	

*OR and CrI are presented. Comparisons with high confidence rating based on CINeMA evaluation are identified in bold. OR higher than 1 favor the intervention specified in the row*.

*NMA, network meta-analysis; PASI, Psoriasis Area Severity Index; CINeMA, Confidence in Network Meta-Analysis; OR, odds ratio; CrI, credible interval; ADA, adalimumab; ETN, etanercept; IFX, infliximab; GOL, golimumab; CZP, certolizumab; UST, ustekinumab; SEC, secukinumab; IXE, ixekizumab; GUS, guselkumab; BRD, brodalumab; RIS, risankizumab; TIL, tildrakizumab; BRK, briakinumab; MTX, methotrexate; PLB, placebo*.

In the ACR70 NMA (Table 4), the results of the comparisons between drugs are not reliable, except when compared with the placebo. From the 12 RCTs reporting ACR70 responses at week 24 (Table 3) (1, 11, 18, 24, 26, 28, 31, 32, 37, 38, 49, 51), only one performed head-to-head comparisons, at week 24, and there was no superiority regarding this specific endpoint (51). The other head-to-head study is EXCEED, with a primary endpoint at 52 weeks, showing also no superiority regarding ACR70. Nevertheless, and as expected, compared with the placebo, all drugs were significantly better in achieving ACR20/50/70.

In the PASI100 response NMA (Table 8), as for ACR70, the comparisons with high confidence levels were few and therefore it was not possible to rank the drugs regarding their probability to achieve differences in PASI100 between weeks 10 and 16. The comparisons with placebo were reliable, and the drugs that lead to a higher probability in achieving PASI100 were BRD, RIS, IXE, and GUS. Although based on CINeMA analysis we were not able to have high confidence in all of our comparisons, the results from placebo comparison were partially following recently published network meta-analysis (94–96). Although the number of RCTs reporting PASI100 response (Table 7) (15, 16, 20, 21, 25, 29, 30, 33, 35–37, 39–41, 43, 44, 46, 48, 51–53, 56, 57, 59, 70) as an outcome was superior to the ones reporting ACR70 response, the confidence in the NMA was not superior. Since 2015 some head-to-head trials were designed to evaluate the efficacy of specific drugs in the PASI response outcome (33, 40, 41, 51–53, 55, 57), and significant differences were found (Table 7).

A complete treatment of a patient with PsD should be ideally based on a single drug that is effective in all the manifestations. Currently, from the therapies included in the PASI100 NMAs, only ADA, CZP, IXE, SEC, and UST were approved for PSO and PsA. Thus, in integrative analysis of NMA results, and based only on comparisons of the drugs with placebo, those with the highest probability of reaching the proposed outcome for skin and joint domains are SEC and IXE. For SEC, OR (95% CrI) are 9.430 (5.455, 16.302) and 42.897 (26.848, 68.539) versus placebo for ACR70 and PASI100, respectively. For IXE, OR are 9.315 (4.206, 20.627) and 64.027 (39.805, 102.997) versus placebo for ACR70 and PASI100, respectively. Even though a few previous NMAs analyzed treatment options in PsD including ACR and PASI outcomes, most of them did not find significant differences in the efficacy and safety between the drugs, only detecting that treatments were more efficacious than placebo (97–101).

As reported in Table 5, data regarding the enthesitis domain were not so consistent as skin and peripheral arthritis results (1, 13, 18, 22, 24, 26, 28, 31, 32, 37, 38, 47, 50, 51, 55, 61, 65, 76). In addition to the outcome not being standardized, there were studies reporting more than one outcome without consistent results (50, 51). There were drugs that even in comparison with the placebo did not show a consistent significant benefit (22, 28, 31, 38, 76). Long-term evaluation of enthesitis showed that the benefit was maintained with IFX at week 54 (58). Although the benefit of UST was not consistent at weeks 24 and 52(28), at week 100 there was a 100% improvement of MASES from baseline (67) and the same was true for SEC results, which showed inconsistent data at week 24 (31, 32), but at week 104 there was 100% resolution of enthesitis in 70% of the patients who had enthesitis at baseline (75). Enthesopathy affects 35–50% of patients with PsA and should be managed carefully since it can affect the quality of life and work productivity even in the early stages of the disease (102). A recent study showed that enthesitis is the phenotypes of PsD that contribute most to Quality of Life Scores and that this domain should be evaluated, bilaterally, in all PsD patients, particularly in those referring joint pain (103). Nevertheless, the clinical evaluation of enthesitis is not standardized and lacks accuracy and the reliability is highly dependent on the observer (104). A recent study compared MASES, SPARCC, and LEI, the three enthesitis index, and showed that MASES had a better correlation with disease activity and functional measures (105). On the other hand, another study has reported a better performance in LEI and SPARCC indices, which showed a higher discriminatory ability and treatment responses suggested to be related to the fact that MASES evaluates fewer peripheral sites, which may be clinically relevant in the context of PsA, a predominantly peripheral disease (106).

Similarly to enthesitis, the outcomes measured in the dactylitis domain were not standardized as is explicit in Table 6 (1, 18, 24, 26, 28, 31, 32, 37, 38, 47, 51, 55, 61, 76). Moreover, there were data with the same drug in different studies that were not consistent (31, 32, 37, 38). Long-term data showed that the benefit with IFX was maintained at week 54 (58). For UST, the median percent improvement in the enthesitis score at week 100 was 100% (67) whereas for SEC treatment 90% of the patients presented complete dactylitis resolution at week 104 (75). A major limitation in dactylitis evaluation is that physical examination is the basis for the clinical assessment of dactylitis and imaging tools have been used only to complement the clinical examination. Nevertheless, the criteria for image resolution are not uniform and therefore data from different studies are not comparable (107, 108). Like enthesitis, dactylitis also has a huge impact on the quality of life and in the structural impact of PsD, and data from enthesitis and dactylitis highlight the difficulty in treating these manifestations and the long period of treatment that is needed to achieve remission. Recently, a real-world PsA population multinational study has shown that enthesitis, dactylitis, inflammatory back pain, and sacroiliitis are significantly associated with the worsening of the patient's quality of life and/or work productivity, through evaluation of an extensive patient-reported outcomes (PROs) list—namely EQ-5D, HAQ-DI, Psoriatic Arthritis Impact of Disease (PsAID)12, and Work Productivity and Activity Impairment (WPAI) (109).

Inflammatory back pain and sacroiliitis are common axial manifestations in PsA patients and can arise in 30 to 70% of patients (110, 111). There is an ongoing discussion on whether axial manifestations in PsA are equivalent to those seen in axial spondyloarthritis and consequently if they may be treated in the same way (112). In fact, the evidence of the efficacy of biologic therapies in the PsA axial domain is still scarce. However, some studies and case reports have suggested a positive impact of TNFi, IL-17i, and IL-12/23i in axial involvement-related outcomes in PsD patients, namely, BASDAI and ASAS-PR, showing that it could be possible to achieve remission and minimal disease activity (113–115). To our knowledge, the only randomized clinical trial addressing treatment efficacy in this specific domain patient profile is the MAXIMIZE trial (ClinicalTrials.gov NCT02721966) (86)—a study evaluating SEC efficacy in axial manifestations improvement in PsA patients. In fact, from the data released in the latest international congresses, results suggest that IL-17 inhibition, namely, with SEC, is effective in axial PsA treatment, evaluated by ASAS response and Berlin MRI score (116).

Nail psoriasis is common among patients with moderate-to-severe PsO and more prevalent in patients with PsA (117). Different studies assessed the efficacy of biologic agents in the treatment and resolution of nail psoriasis (Table 9) (12, 18, 26, 27, 34, 37, 38, 42, 46, 51, 61, 65, 69, 73, 77, 78, 81, 88). All of them showed the benefit of the tested drug compared to the placebo. The head-to-head comparison between IXE and ADA showed superiority at week 24 of IXE (51). The response is sustained in long-term studies (46, 69, 81). Of note, most studies reporting NAPSI represent subgroup analysis including recruited patients who had manifestations of nail psoriasis. However, from the data described there are only drugs with studies designed specifically to evaluate nail Psoriasis: ETN (89), ADA (42), and SEC (46). Importantly, these studies were specifically designed to evaluate nail outcomes and have demanding recruitment criteria, with NAPSI scores more severe and, therefore, much more difficult to treat. Therefore, the results obtained with these 3 drugs may be considered more robust and significant concerning their impact on nail treatment. Of note, all studies demonstrated an improvement in the evaluated scores. However, scores and time points were not the same, making comparisons impossible.

**Table 9:** Nail psoriasis assessment in patients with psoriasis.

**Study**	**Author**	**Year**	**Intervention**	**Outcome**	**Time of outcome**	**Result**
(12)	Reich	2005	IFX vs. placebo	Percentage of improvement NAPSI	Week 10	26.0 (42.3) vs. −5.9 (54.3) (*p <* 0.0001)
					Week 24	56.3 (43.3) vs. −3.2 (62.3) (*p <* 0.0001)
(18)	Kavanaugh	2009	GOL 50 mg vs. placebo	Percentage of change NAPSI	Week 14	25% vs. 0% (*p =* 0.015)
			GOL 100 mg vs. placebo			53% vs. 0% (*p <* 0.001)
			GOL 50 mg vs. placebo		Week 24	33% vs. 0% (*p <* 0.001)
			GOL 100 mg vs. placebo			54% vs. 0% (*p <* 0.001)
GO-REVEAL (61)	Kavanaugh	2012	GOL 50 mg vs. placebo	NAPSI (percentage change from baseline)	Week 52	51.6 ± 46.8 vs. 56.2 ± 48.1
			GOL 100 mg vs. placebo			65.8 ± 51.9 vs. 56.2 ± 48.1
	Ortonne	2013	ETN 50 mg BIW	NAPSI	Week 12	−13.6 (−16.7 to −10.5)
			ETN 50 mg QW			−15.7 (−19,0 to −12.5)
PHOENIX 1 (27)	Rich	2014	UST 45 mg vs. placebo	NAPSI baseline score	Week 12	26.7% vs. 11.8% (*p <* 0.001)
			UST 90 mg vs. placebo			24.9% vs. 11.8% (*p <* 0.001)
			UST 45 mg vs. placebo		Week 24	46.5% vs. 29.1%
(65)	Kavanaugh	2014	GOL 50 mg vs. placebo	NAPSI	Week 256	1.7 ± 2.5 vs. 1.1 ± 1.9
			GOL 100 mg vs. placebo			1.1 ± 1.8 vs. 1.1 ± 1.9
BELIEVE (33)	Thaci	2015	ADA	NAPSI baseline reduction	Week 8	15.1%
					Week 16	39.5%
(26)	Mease	2015	CZP 200 mg Q2W vs. placebo	mNAPSI change from baseline	Week24	– 1.6 VS. −1.1 (*p =* 0.003)
			CZP 400 mg Q4W vs. placebo			−2.0 vs. −1.1 (*p <* 0.001)
UNCOVER 3 (69)	Dennehy	2016	IXE Q2W vs. placebo vs. ETN	Improvement in nail psoriasis	Week 12	38% vs. 28% vs. −4.7%
			IXE Q4W vs. placebo vs. ETN			40% vs. 48% vs. −4,7%
SPIRIT-P1 (37)	Mease	2017	IXE Q2W vs. placebo vs. ADA	NAPSI	Week 12	−7.7 (1.4) vs. −1.1 (1.4) vs. −6.8 (1.4) *p <* 0.05*
			IXE Q4W vs. placebo vs. ADA			−15.5 (1.5) vs. −2.4 (1.7) vs. −10.7 (1.5) *p <* 0.05*
			IXE Q2W vs. placebo vs. ADA		Week 24	−8.4 (1.5) vs. −1.1 (1.4) vs. −6.8 (1.4) *p <* 0.05*
			IXE Q4W vs. placebo vs. ADA			−14.0 (1.5) 2.4 (1.7) vs. −10.7 (1.5) *p <* 0.05*
SPIRIT-P2 (38)	Nash	2017	IXE Q2W vs. placebo	Proportion of patients who had a response	Week 24	34.8% vs. 11.0% (*p <* 0.0005)
			IXE Q4W vs. placebo			20% vs. 7.0% (*p <* 0.0001)
UNCOVER 3 (78)	van der Kerkhof	2017	IXE Q2W vs. placebo	NAPSI from baseline	Week 12	35.2% vs. −34.3% *p <* 0.001
			IXE Q4W vs. placebo			36.7% vs. −34.3% *p <* 0.001
			IXE Q2W vs. ETN			35.2 (6.9) vs. 20.0 (5.9) *p >* 0.005
			IXE Q4W vs. ETN			36.7% vs. 20% *p =* 0.048
			IXE Q2W vs. placebo	NAPSI = 0	Week 12	17.5% vs. 4.3% *p <* 0.001
			IXE Q4W vs. placebo			19.7% vs. 4.3% *p <* 0.001
			IXE Q2W vs. ETN			17.5% vs. 10.2% *p <* 0.05
			IXE Q4W vs. ETN			19.7% vs. 10.2% *p <* 0.05
(36)	Blauvelt	2017	GUS vs. placebo vs. ADA	NAPSI percent improvement	Week 16	34.4 ± 42.46 vs. −0.9 ± 57.89 vs. 38.0 ± 53.87 *p <* 0.001**
			GUS vs. ADA		Week 24	49.8 ± 44.16 vs. 49.4 ± 60.04
			GUS vs. ADA		Week 48	68.1 ± 43.00 vs. 61.4 ± 49.20
LIBERATE (77)	Reich	2017	APR vs. placebo	NAPSI (percentage of change)	Week 16	−18.7 (40.2) vs. −17.0 (25.0) *p =* 0.4959
			ETN vs. placebo			−35.9 (28.9) vs. −17.0 (25.0) *p =* 0.0024
(42)	Elewski	2018	ADA vs. placebo	mNAPSI75	Week 26	46.6% vs. 3.4% (*p <* 0.001)
				Improvement NAPSI	Week 26	56.2% vs. 11.5% (*p <* 0.01)
			UST 90 mg vs. placebo			48.7% vs. 29.1%
(81)	Ohtsuki	2018	GUS 50 mg vs. placebo	Change in NAPSI	Week 16	−1.2 (1.61) vs. −0.2 (1.13) *p <* 0.001
			GUS 100 mg vs. placebo			−1.5 (1.78) vs. −0.2 (1.13) *p <* 0.001
TRANSFIGURE (46)	Reich	2018	SEC 150 mg vs. placebo	NAPSI (percentage of change)	Week 16	−37.9% vs. −10.8% (*p <* 0.001)
			SEC 300 mg vs. placebo			−45.3% vs. −10.8% (*p <* 0.001)
(88)	Elewski	2019	ADA	Percentage of achievement mNAPSI75	Week 16	27.3
					Week 26	53.4
					Week 52	65.0
SPIRIT H2H (51)	Mease	2020	IXE vs. ADA	Fingernails NAPSI = 0	Week 24	58.1% vs. 71.7% (*p <* 0.001)

ADA, adalimumab; ETN, etanercept; IFX, infliximab; GOL, golimumab; CZP, certolizumab; UST, ustekinumab; SEC, secukinumab; IXE, ixekizumab; GUS, guselkumab; RIS, risankizumab; PLB, placebo; NAPSI, Nail Psoriasis Severity Index; mNAPSI, modified Nail Psoriasis Index. *IXE vs. placebo, **GUS vs. placebo.

Taking all the results from the systematic review and network meta-analysis together in Figure 2, IL-17i are the drugs tested in more manifestations, namely, SEC that had specific studies for all the domains, even though axial domain data were not yet published.

This result is in line with what was recently published in two NMA (98, 118). The first one concluded that SEC demonstrated good efficacy across the evaluated outcomes (ACR, PASI, and PsARC at 12–16 weeks) and all the treatments demonstrated superiority to placebo (98). The other study demonstrated that SEC may be the most efficacious and the safest biologic for short-term treatment of PsA (118).

### Limitations

One of the main limitations of this study is the high variability of study designs, inclusion and exclusion criteria, and patients' characteristics. It is important to note that for enthesitis, dactylitis, and nail psoriasis the evaluated outcomes are heterogeneous and do not allow the performance of a network meta-analysis. The results of the NMAs highlight the limitations of this method, and caution is needed in the interpretation of these results to avoid misleading inferences.

## Conclusions

PsD is a very complex disease in which the same patient may present several manifestations with a great impact on functional and quality of life. Nowadays, we should be more demanding in the analysis of therapeutic outcomes, focusing on achieving remission in all PsD manifestations.

Although there are several effective therapies, this study showed that the concept of a holistic and efficacious treatment for patients with PsD is achievable and that IL-17i are the drugs most extensively tested in this context. Specifically, SEC demonstrated good efficacy in all the evaluated GRAPPA domains, allowing a complete short-term treatment for patients with multiple manifestations of the disease.

## Data Availability Statement

The original contributions presented in the study are included in the article/supplementary materials, further inquiries can be directed to the corresponding author/s.

## Author Contributions

TT and AB conceptualized the study, designed PICO criteria, managed the literature search, and wrote the first draft of the manuscript. PF and JF interpreted the data and critically revised the manuscript. All the authors approved the final manuscript.

## Conflict of Interest

TT has received research grants and/or consulting fees from AbbVie, Almirall, Amgen, Arena Pharmaceuticals, Biocad, Biogen, Boehringer Ingelheim, Bristol-Myers Squibb, Celgene, Eli Lilly Janssen, LEO Pharma, MSD, Novartis, Pfizer, Samsung-Bioepis, Sandoz, and Sanofi. AB has received consulting grants or acted as a speaker for Novartis, MSD, Eli-Lilly, Abbvie, Bene, and Pfizer. JF has received unrestricted research grants or acted as a speaker for Abbvie, Ache, Amgen, BIAL, Biogen, BMS, Janssen, Lilly, MSD, Novartis, Pfizer, Roche, Sanofi, and UCB. The remaining author declares that the research was conducted in the absence of any commercial or financial relationships that could be construed as a potential conflict of interest.

## References

[1] Antoni C Krueger GG de Vlam K Birbara C Beutler A Guzzo C. Infliximab improves signs and symptoms of psoriatic arthritis: results of the IMPACT 2 trial. Ann Rheumat Dis. (2005) 64:1150–7. 10.1136/ard.2004.032268 15677701 PMC1755609

[2] Coates LC Kavanaugh A Mease PJ Soriano ER Laura Acosta-Felquer M Armstrong AW. Group for research and assessment of psoriasis and psoriatic arthritis 2015. Treatment recommendations for psoriatic arthritis. Arthr Rheumatol. (2016) 68:1060–71. 10.1002/art.39573 26749174

[3] Scarpa R Caso F Costa L Peluso R Del Puente A Olivieri I. Psoriatic disease 10 years later. J Rheumatol. (2017) 44:1298–301. 10.3899/jrheum.161402 28864664

[4] Sakkas LI Zafiriou E Bogdanos DP. Mini review: new treatments in psoriatic arthritis. Focus on the IL-23/17 Axis. Front Pharmacol. (2019) 10:872. 10.3389/fphar.2019.00872 31447673 PMC6691125

[5] Chimenti MS Caso F Alivernini S De Martino E Costa L Tolusso B. Amplifying the concept of psoriatic arthritis: the role of autoimmunity in systemic psoriatic disease. Autoimmun Rev. (2019) 18:565–75. 10.1016/j.autrev.2018.11.007 30959209

[6] Raychaudhuri SP Wilken R Sukhov AC Raychaudhuri SK Maverakis E. Management of psoriatic arthritis: early diagnosis, monitoring of disease severity and cutting edge therapies. J Autoimmun. (2017) 76:21–37. 10.1016/j.jaut.2016.10.009 27836567

[7] [Software] CCiNM-A. Institute of Social and Preventive Medicine, University of Bern (2017).

[8] Salanti G Del Giovane C Chaimani A Caldwell DM Higgins JPT Evaluating the quality of evidence from a network meta-analysis. PLoS ONE. (2014) 9:e99682 10.1371/journal.pone.0099682 24992266 PMC4084629

[9] Current Version of RoB 2 (2019). Available online at: https://sites.google.com/site/riskofbiastool/welcome/rob-2-0-tool/current-version-of-rob-2 (accessed July 15, 2020).

[10] Moher D Liberati A Tetziaff J Altman DG The PRISMA Group (2009). Preferred reporting items for systematic reviews and meta-analysis: the PRISMA statement. PLoS Med. 6:e1000097 10.137/journal.pmed1000097 19621072 PMC2707599

[11] Mease PJ Gladman DD Ritchlin CT Ruderman EM Steinfeld SD Choy EH. Adalimumab for the treatment of patients with moderately to severely active psoriatic arthritis: results of a double-blind, randomized, placebo-controlled trial. Arthr Rheumat. (2005) 52:3279–89. 10.1002/art.21306 16200601

[12] Reich K Nestle FO Papp K Ortonne JP Evans R Guzzo C. Infliximab induction and maintenance therapy for moderate-to-severe psoriasis: a phase III, multicentre, double-blind trial. Lancet. (2005) 366:1367–74. 10.1016/S0140-6736(05)67566-6 16226614

[13] Genovese MC Mease PJ Thomson GT Kivitz AJ Perdok RJ Weinberg MA. Safety and efficacy of adalimumab in treatment of patients with psoriatic arthritis who had failed disease modifying antirheumatic drug therapy. J Rheumatol. (2007) 34:1040–50. 17444593

[14] Tyring S Gordon KB Poulin Y Langley RG Gottlieb AB Dunn M. Long-term safety and efficacy of 50 mg of etanercept twice weekly in patients with psoriasis. Arch Dermatol. (2007) 143:719–26. 10.1001/archderm.143.6.719 17576937

[15] Leonardi CL Kimball AB Papp KA Yeilding N Guzzo C Wang Y. Efficacy and safety of ustekinumab, a human interleukin-12/23 monoclonal antibody, in patients with psoriasis: 76-week results from a randomised, double-blind, placebo-controlled trial (PHOENIX 1). Lancet. (2008) 371:1665–74. 10.1016/S0140-6736(08)60725-4 18486739

[16] Papp KA Langley RG Lebwohl M Krueger GG Szapary P Yeilding N. Efficacy and safety of ustekinumab, a human interleukin-12/23 monoclonal antibody, in patients with psoriasis: 52-week results from a randomised, double-blind, placebo-controlled trial (PHOENIX 2). Lancet. (2008) 371:1675–84. 10.1016/S0140-6736(08)60726-6 18486740

[17] Rich P Griffiths CE Reich K Nestle FO Scher RK Li S. Baseline nail disease in patients with moderate to severe psoriasis and response to treatment with infliximab during 1 year. J Am Acad Dermatol. (2008) 58:224–31. 10.1016/j.jaad.2007.07.042 18083272

[18] Kavanaugh A McInnes I Mease P Krueger GG Gladman D Gomez-Reino J. Golimumab, a new human tumor necrosis factor alpha antibody, administered every four weeks as a subcutaneous injection in psoriatic arthritis: twenty-four-week efficacy and safety results of a randomized, placebo-controlled study. Arthr Rheumat. (2009) 60:976–86. 10.1002/art.24403 19333944

[19] Barker J Hoffmann M Wozel G Ortonne JP Zheng H van Hoogstraten H. Efficacy and safety of infliximab vs. methotrexate in patients with moderate-to-severe plaque psoriasis: results of an open-label, active-controlled, randomized trial (RESTORE1). Br J Dermatol. (2011) 165:1109–17. 10.1111/j.1365-2133.2011.10615.x 21910713

[20] Gottlieb AB Leonardi C Kerdel F Mehlis S Olds M Williams DA. Efficacy and safety of briakinumab vs. etanercept and placebo in patients with moderate to severe chronic plaque psoriasis. Br J Dermatol. (2011) 165:652–60. 10.1111/j.1365-2133.2011.10418.x 21574983

[21] Strober BE Crowley JJ Yamauchi PS Olds M Williams DA. Efficacy and safety results from a phase III, randomized controlled trial comparing the safety and efficacy of briakinumab with etanercept and placebo in patients with moderate to severe chronic plaque psoriasis. Br J Dermatol. (2011) 165:661–8. 10.1111/j.1365-2133.2011.10419.x 21574984

[22] Baranauskaite A Raffayova H Kungurov NV Kubanova A Venalis A Helmle L. Infliximab plus methotrexate is superior to methotrexate alone in the treatment of psoriatic arthritis in methotrexate-naive patients: the RESPOND study. Ann Rheum Dis. (2012) 71:541–8. 10.1136/ard.2011.152223 21994233 PMC3298666

[23] Gottlieb AB Langley RG Strober BE Papp KA Klekotka P Creamer K. A randomized, double-blind, placebo-controlled study to evaluate the addition of methotrexate to etanercept in patients with moderate to severe plaque psoriasis. Br J Dermatol. (2012) 167:649–57. 10.1111/j.1365-2133.2012.11015.x 22533447 PMC3504074

[24] McInnes IB Kavanaugh A Gottlieb AB Puig L Rahman P Ritchlin C. Efficacy and safety of ustekinumab in patients with active psoriatic arthritis: 1 year results of the phase 3, multicentre, double-blind, placebo-controlled PSUMMIT 1 trial. Lancet. (2013) 382:780–9. 10.1016/S0140-6736(13)60594-2 23769296

[25] Langley RG Elewski BE Lebwohl M Reich K Griffiths CE Papp K. Secukinumab in plaque psoriasis–results of two phase 3 trials. N Engl J Med. (2014) 371:326–38. 10.1056/NEJMoa1314258 25007392

[26] Mease PJ Fleischmann R Deodhar AA Wollenhaupt J Khraishi M Kielar D. Effect of certolizumab pegol on signs and symptoms in patients with psoriatic arthritis: 24-week results of a Phase 3 double-blind randomised placebo-controlled study (RAPID-PsA). Ann Rheum Dis. (2014) 73:48–55. 10.1136/annrheumdis-2013-203696 23942868 PMC3888622

[27] Rich P Bourcier M Sofen H Fakharzadeh S Wasfi Y Wang Y. Ustekinumab improves nail disease in patients with moderate-to-severe psoriasis: results from PHOENIX 1. Br J Dermatol. (2014) 170:398–407. 10.1111/bjd.12632 24117389

[28] Ritchlin C Rahman P Kavanaugh A McInnes IB Puig L Li S. Efficacy and safety of the anti-IL-12/23 p40 monoclonal antibody, ustekinumab, in patients with active psoriatic arthritis despite conventional non-biological and biological anti-tumour necrosis factor therapy: 6-month and 1-year results of the phase 3, multicentre, double-blind, placebo-controlled, randomised PSUMMIT 2 trial. Ann Rheum Dis. (2014) 73:990–9. 10.1136/annrheumdis-2013-204655 24482301 PMC4033144

[29] Griffiths CE Reich K Lebwohl M van de Kerkhof P Paul C Menter A Comparison of ixekizumab with etanercept or placebo in moderate-to-severe psoriasis (UNCOVER-2 and UNCOVER-3): results from two phase 3 randomised trials. Lancet. (2015) 386:541–51. 10.1016/S0140-6736(15)60125-8 26072109

[30] Lebwohl M Strober B Menter A Gordon K Weglowska J Puig L. Phase 3 studies comparing brodalumab with ustekinumab in psoriasis. N Engl J Med. (2015) 373:1318–28. 10.1056/NEJMoa1503824 26422722

[31] McInnes IB Mease PJ Kirkham B Kavanaugh A Ritchlin CT Rahman P. Secukinumab, a human anti-interleukin-17A monoclonal antibody, in patients with psoriatic arthritis (FUTURE 2): a randomised, double-blind, placebo-controlled, phase 3 trial. Lancet. (2015) 386:1137–46. 10.1016/S0140-6736(15)61134-5 26135703

[32] Mease PJ McInnes IB Kirkham B Kavanaugh A Rahman P van der Heijde D. Secukinumab inhibition of interleukin-17a in patients with psoriatic arthritis. N Engl J Med. (2015) 373:1329–39. 10.1056/NEJMoa1412679 26422723

[33] Thaçi D Blauvelt A Reich K Tsai TF Vanaclocha F Kingo K Secukinumab is superior to ustekinumab in clearing skin of subjects with moderate to severe plaque psoriasis: CLEAR, a randomized controlled trial. J Am Acad Dermatol. (2015) 73:400–9. 10.1016/j.jaad.2015.05.013 26092291

[34] Thaçi D Unnebrink K Sundaram M Sood S Yamaguchi Y. Adalimumab for the treatment of moderate to severe psoriasis: subanalysis of effects on scalp and nails in the BELIEVE study. J Eur Acad Dermatol Venereol. (2015) 29:353–60. 10.1111/jdv.12553 24846518

[35] Papp KA Reich K Paul C Blauvelt A Baran W Bolduc C. A prospective phase III, randomized, double-blind, placebo-controlled study of brodalumab in patients with moderate-to-severe plaque psoriasis. Br J Dermatol. (2016) 175:273–86. 10.1111/bjd.14493 26914406

[36] Blauvelt A Papp KA Griffiths CE Randazzo B Wasfi Y Shen YK. Efficacy and safety of guselkumab, an anti-interleukin-23 monoclonal antibody, compared with adalimumab for the continuous treatment of patients with moderate to severe psoriasis: results from the phase III, double-blinded, placebo- and active comparator-controlled VOYAGE 1 trial. J Am Acad Dermatol. (2017) 76:405–17. 10.1016/j.jaad.2016.11.041 28057360

[37] Mease PJ van der Heijde D Ritchlin CT Okada M Cuchacovich RS Shuler CL. Ixekizumab, an interleukin-17A specific monoclonal antibody, for the treatment of biologic-naive patients with active psoriatic arthritis: results from the 24-week randomised, double-blind, placebo-controlled and active (adalimumab)-controlled period of the phase III trial SPIRIT-P1. Ann Rheum Dis. (2017) 76:79–87. 10.1136/annrheumdis-2016-209709 27553214 PMC5264219

[38] Nash P Kirkham B Okada M Rahman P Combe B Burmester GR. Ixekizumab for the treatment of patients with active psoriatic arthritis and an inadequate response to tumour necrosis factor inhibitors: results from the 24-week randomised, double-blind, placebo-controlled period of the SPIRIT-P2 phase 3 trial. Lancet. (2017) 389:2317–7. 10.1136/annrheumdis-2017-eular.1576 28551073

[39] Reich K Papp KA Blauvelt A Tyring SK Sinclair R Thaci D. Tildrakizumab versus placebo or etanercept for chronic plaque psoriasis (reSURFACE 1 and reSURFACE 2): results from two randomised controlled, phase 3 trials. Lancet. (2017) 390:276–88. 10.1016/S0140-6736(17)31279-5 28596043

[40] Reich K Pinter A Lacour JP Ferrandiz C Micali G French LE. Comparison of ixekizumab with ustekinumab in moderate-to-severe psoriasis: 24-week results from IXORA-S, a phase III study. Br J Dermatol. (2017) 177:1014–23. 10.1111/bjd.15666 28542874

[41] Bagel J Nia J Hashim PW Patekar M de Vera A Hugot S. Secukinumab is superior to ustekinumab in clearing skin in patients with moderate to severe plaque psoriasis (16-week clarity results). Dermatol Ther. (2018) 8:571–9. 10.1007/s13555-018-0265-y 30334147 PMC6261116

[42] Elewski BE Okun MM Papp K Baker CS Crowley JJ Guillet G. Adalimumab for nail psoriasis: efficacy and safety from the first 26 weeks of a phase 3, randomized, placebo-controlled trial. J Am Acad Dermatol. (2018) 78:90–9.e1. 10.1016/j.jaad.2017.08.029 28993005

[43] Gordon KB Strober B Lebwohl M Augustin M Blauvelt A Poulin Y. Efficacy and safety of risankizumab in moderate-to-severe plaque psoriasis (UltIMMa-1 and UltIMMa-2): results from two double-blind, randomised, placebo-controlled and ustekinumab-controlled phase 3 trials. Lancet. (2018) 392:650–61. 10.1016/S0140-6736(18)31713-6 30097359

[44] Gottlieb AB Blauvelt A Thaci D Leonardi CL Poulin Y Drew J. Certolizumab pegol for the treatment of chronic plaque psoriasis: results through 48 weeks from 2 phase 3, multicenter, randomized, double-blinded, placebo-controlled studies (CIMPASI-1 and CIMPASI-2). J Am Acad Dermatol. (2018) 79:302–14.e6. 10.1016/j.jaad.2018.04.012 29660421

[45] Lebwohl M Blauvelt A Paul C Sofen H Weglowska J Piguet V. Certolizumab pegol for the treatment of chronic plaque psoriasis: results through 48 weeks of a phase 3, multicenter, randomized, double-blind, etanercept- and placebo-controlled study (CIMPACT). J Am Acad Dermatol. (2018) 79:266–76.e5. 10.1016/j.jaad.2018.04.013 29660425

[46] Reich K Sullivan J Arenberger P Mrowietz U Jazayeri S Augustin M Effect of secukinumab on the clinical activity and disease burden of nail psoriasis: 32-week results from the randomized placebo-controlled TRANSFIGURE trial. Br J Mermatol. (2018) 181:954–66. 10.1111/bjd.17351 30367462

[47] Mease P van der Heijde D Landewé R Mpofu S Rahman P Tahir H. Secukinumab improves active psoriatic arthritis symptoms and inhibits radiographic progression: primary results from the randomised, double-blind, phase III FUTURE 5 study. Ann Rheumatic Dis. (2018) 77:890–7. 10.1136/annrheumdis-2017-212687 29550766 PMC5965348

[48] Ohtsuki M Fujita H Watanabe M Suzaki K Flack M Huang X. Efficacy and safety of risankizumab in Japanese patients with moderate to severe plaque psoriasis: results from the SustaIMM phase 2/3 trial. J Dermatol. (2019) 46:686–94. 10.1111/1346-8138.14941 31237727 PMC6771602

[49] Mease PJ Rahman P Gottlieb AB Kollmeier AP Hsia EC Xu XL. Guselkumab in biologic-naive patients with active psoriatic arthritis (DISCOVER-2): a double-blind, randomised, placebo-controlled phase 3 trial. Lancet. (2020) 395:1126–36. 10.1016/S0140-6736(20)30263-4 32178766

[50] Araujo EG Englbrecht M Hoepken S Finzel S Kampylafka E Kleyer A. Effects of ustekinumab versus tumor necrosis factor inhibition on enthesitis: results from the enthesial clearance in psoriatic arthritis (ECLIPSA) study. Semin Arthr Rheumat. (2019) 48:632–7. 10.1016/j.semarthrit.2018.05.011 30037432

[51] Mease PJ Smolen JS Behrens F Nash P Liu Leage S Li L. A head-to-head comparison of the efficacy and safety of ixekizumab and adalimumab in biological-naïve patients with active psoriatic arthritis: 24-week results of a randomised, open-label, blinded-assessor trial. Ann Rheum Dis. (2020) 79:123–31. 10.1136/annrheumdis-2019-215386 31563894 PMC6937408

[52] Reich K Gooderham M Thaçi D Crowley JJ Ryan C Krueger JG. Risankizumab compared with adalimumab in patients with moderate-to-severe plaque psoriasis (IMMvent): a randomised, double-blind, active-comparator-controlled phase 3 trial. Lancet. (2019) 394:576–86. 10.1016/S0140-6736(19)30952-3 31280967

[53] Reich K Armstrong AW Langley RG Flavin S Randazzo B Li S. Guselkumab versus secukinumab for the treatment of moderate-to-severe psoriasis (ECLIPSE): results from a phase 3, randomised controlled trial. Lancet. (2019) 394:831–9. 10.1016/S0140-6736(19)31773-8 31402114

[54] Deodhar A Helliwell PS Boehncke WH Kollmeier AP Hsia EC Subramanian RA. Guselkumab in patients with active psoriatic arthritis who were biologic-naive or had previously received TNFα inhibitor treatment (DISCOVER-1): a double-blind, randomised, placebo-controlled phase 3 trial. Lancet. (2020) 395:1115–25. 10.1016/S0140-6736(20)30265-8 32178765

[55] McInnes IB Behrens F Mease PJ Kavanaugh A Ritchlin C Nash P. Secukinumab versus adalimumab for treatment of active psoriatic arthritis (EXCEED): a double-blind, parallel-group, randomised, active-controlled, phase 3b trial. Lancet. (2020) 395:1496–505. 10.1016/S0140-6736(20)30564-X 32386593

[56] Ferris LK Ott E Jiang J Hong HC Li S Han C. Efficacy and safety of guselkumab, administered with a novel patient-controlled injector (One-Press), for moderate-to-severe psoriasis: results from the phase 3 ORION study. J Dermatol Treat. (2020) 31:152–9. 10.1080/09546634.2019.1587145 30887876

[57] Warren RB Blauvelt A Poulin Y Beeck S Kelly M Wu T Efficacy and safety of risankizumab vs. secukinumab in patients with moderate-to-severe plaque psoriasis (IMMerge): results from a phase 3, randomised, open-label, efficacy assessor-blinded clinical trial. Br J Dermatol. (2020). 10.1111/bjd.19341. [Epub ahead of print]. PMC7983954 32594522

[58] Kavanaugh A Krueger GG Beutler A Guzzo C Zhou B Dooley LT. Infliximab maintains a high degree of clinical response in patients with active psoriatic arthritis through 1 year of treatment: results from the IMPACT 2 trial. Ann Rheum Dis. (2007) 66:498–505. 10.1136/ard.2006.058339 17114188 PMC1856065

[59] Menter A Tyring SK Gordon K Kimball AB Leonardi CL Langley RG. Adalimumab therapy for moderate to severe psoriasis: a randomized, controlled phase III trial. J Am Acad Dermatol. (2008) 58:106–15. 10.1016/j.jaad.2007.09.010 17936411

[60] Gordon K Papp K Poulin Y Gu Y Rozzo S Sasso EH. Long-term efficacy and safety of adalimumab in patients with moderate to severe psoriasis treated continuously over 3 years: results from an open-label extension study for patients from REVEAL. J Am Acad Dermatol. (2012) 66:241–51. 10.1016/j.jaad.2010.12.005 21752491

[61] Kavanaugh A van der Heijde D McInnes IB Mease P Krueger GG Gladman DD. Golimumab in psoriatic arthritis: one-year clinical efficacy, radiographic, and safety results from a phase III, randomized, placebo-controlled trial. Arthr Rheumat. (2012) 64:2504–17. 10.1002/art.34436 22378566

[62] Kimball AB Gordon KB Fakharzadeh S Yeilding N Szapary PO Schenkel B. Long-term efficacy of ustekinumab in patients with moderate-to-severe psoriasis: results from the PHOENIX 1 trial through up to 3 years. Br J Dermatol. (2012) 166:861–72. 10.1111/j.1365-2133.2012.10901.x 22356258

[63] Kavanaugh A McInnes IB Krueger GG Gladman D Beutler A Gathany T. Patient-reported outcomes and the association with clinical response in patients with active psoriatic arthritis treated with golimumab: findings through 2 years of a phase III, multicenter, randomized, double-blind, placebo-controlled trial. Arthr Care Res. (2013) 65:1666–73. 10.1002/acr.22044 23666608 PMC4282022

[64] Kimball AB Papp KA Wasfi Y Chan D Bissonnette R Sofen H. Long-term efficacy of ustekinumab in patients with moderate-to-severe psoriasis treated for up to 5 years in the PHOENIX 1 study. J Eur Acad Dermatol Venereol. (2013) 27:1535–45. 10.1111/jdv.12046 23279003

[65] Kavanaugh A McInnes IB Mease P Krueger GG Gladman D van der Heijde D. Clinical efficacy, radiographic and safety findings through 5 years of subcutaneous golimumab treatment in patients with active psoriatic arthritis: results from a long-term extension of a randomised, placebo-controlled trial (the GO-REVEAL study). Ann Rheum Dis. (2014) 73:1689–94. 10.1136/annrheumdis-2013-204902 24748630 PMC4145441

[66] Kavanaugh A Ritchlin C Rahman P Puig L Gottlieb AB Li S. Ustekinumab, an anti-IL-12/23 p40 monoclonal antibody, inhibits radiographic progression in patients with active psoriatic arthritis: results of an integrated analysis of radiographic data from the phase 3, multicentre, randomised, double-blind, placebo-controlled PSUMMIT-1 and PSUMMIT-2 trials. Ann Rheum Dis. (2014) 73:1000–6. 10.1136/annrheumdis-2013-204741 24553909 PMC4033146

[67] Kavanaugh A Puig L Gottlieb AB Ritchlin C Li S Wang Y. Maintenance of clinical efficacy and radiographic benefit through two years of ustekinumab therapy in patients with active psoriatic arthritis: results from a randomized, placebo-controlled phase III trial. Arthr Care Res. (2015) 67:1739–49. 10.1002/acr.22645 26097039 PMC5063124

[68] Langley RG Lebwohl M Krueger GG Szapary PO Wasfi Y Chan D. Long-term efficacy and safety of ustekinumab, with and without dosing adjustment, in patients with moderate-to-severe psoriasis: results from the PHOENIX 2 study through 5 years of follow-up. Br J Dermatol. (2015) 172:1371–83. 10.1111/bjd.13469 25307931

[69] Dennehy EB Zhang L Amato D Goldblum O Rich P. Ixekizumab is effective in subjects with moderate to severe plaque psoriasis with significant nail involvement: results from UNCOVER 3. J Drugs Dermatol. (2016) 15:958–61. 27537996

[70] Gordon KB Blauvelt A Papp KA Langley RG Luger T Ohtsuki M Phase 3 trials of ixekizumab in moderate-to-severe plaque psoriasis. N Engl J Med. (2016) 375:345–56. 10.1056/NEJMoa1512711 27299809

[71] van der Heijde D Landewe RB Mease PJ McInnes IB Conaghan PG Pricop L. Brief report: secukinumab provides significant and sustained inhibition of joint structural damage in a phase III study of active psoriatic arthritis. Arthr Rheumatol. (2016) 68:1914–21. 10.1002/art.39685 27014997 PMC5129532

[72] Blauvelt A Ferris LK Yamauchi PS Qureshi A Leonardi CL Farahi K. Extension of ustekinumab maintenance dosing interval in moderate-to-severe psoriasis: results of a phase IIIb, randomized, double-blinded, active-controlled, multicentre study (PSTELLAR). Br J Dermatol. (2017) 177:1552–61. 10.1111/bjd.15722 28600818

[73] Blauvelt A Gooderham M Iversen L Ball S Zhang L Agada NO. Efficacy and safety of ixekizumab for the treatment of moderate-to-severe plaque psoriasis: results through 108 weeks of a randomized, controlled phase 3 clinical trial (UNCOVER-3). J Am Acad Dermatol. (2017) 77:855–62. 10.1016/j.jaad.2017.06.153 28917383

[74] Blauvelt A Reich K Tsai TF Tyring S Vanaclocha F Kingo K. Secukinumab is superior to ustekinumab in clearing skin of subjects with moderate-to-severe plaque psoriasis up to 1 year: results from the CLEAR study. J Am Acad Dermatol. (2017) 76:60–9.e9. 10.1016/j.jaad.2016.08.008 27663079

[75] McInnes IB Mease PJ Ritchlin CT Rahman P Gottlieb AB Kirkham B. Secukinumab sustains improvement in signs and symptoms of psoriatic arthritis: 2 year results from the phase 3 FUTURE 2 study. Rheumatology. (2017) 56:1993–2003. 10.1093/rheumatology/kex301 28968735 PMC5850284

[76] Mease P Hall S FitzGerald O van der Heijde D Merola JF Avila-Zapata F Tofacitinib or adalimumab versus placebo for psoriatic arthritis. N Engl J Med. (2017) 377:1537–50. 10.1056/NEJMoa1615975 29045212

[77] Reich K Gooderham M Green L Bewley A Zhang Z Khanskaya I. The efficacy and safety of apremilast, etanercept and placebo in patients with moderate-to-severe plaque psoriasis: 52-week results from a phase IIIb, randomized, placebo-controlled trial (LIBERATE). J Eur Acad Dermatol Venereol. (2017) 31:507–17. 10.1111/jdv.14015 27768242 PMC5363370

[78] van de Kerkhof P Guenther L Gottlieb AB Sebastian M Wu JJ Foley P. Ixekizumab treatment improves fingernail psoriasis in patients with moderate-to-severe psoriasis: results from the randomized, controlled and open-label phases of UNCOVER-3. J Eur Acad Dermatol Venereol. (2017) 31:477–82. 10.1111/jdv.14033 27910156

[79] Griffiths CEM Papp KA Kimball AB Randazzo B Song M Li S. Long-Term efficacy of guselkumab for the treatment of moderate-to-severe psoriasis: results from the phase 3 Voyage 1 trial through two years. J Drugs Dermatol. (2018) 17:826–32. 10.1136/annrheumdis-2018-eular.5825 30124721

[80] Leonardi C Maari C Philipp S Goldblum O Zhang L Burkhardt N. Maintenance of skin clearance with ixekizumab treatment of psoriasis: three-year results from the UNCOVER-3 study. J Am Acad Dermatol. (2018) 79:824–30.e2. 10.1016/j.jaad.2018.05.032 29803904

[81] Ohtsuki M Kubo H Morishima H Goto R Zheng R Nakagawa H. Guselkumab, an anti-interleukin-23 monoclonal antibody, for the treatment of moderate to severe plaque-type psoriasis in Japanese patients: efficacy and safety results from a phase 3, randomized, double-blind, placebo-controlled study. J Dermatol. (2018) 45:1053–62. 10.1111/1346-8138.14504 29905383 PMC6175099

[82] Reich K Gooderham M Bewley A Green L Soung J Petric R Safety and efficacy of apremilast through 104 weeks in patients with moderate to severe psoriasis who continued on apremilast or switched from etanercept treatment: findings from the LIBERATE study. J Eur Acad Dermatol Venereol. (2018) 32:397–402. 10.1111/jdv.14738 29220542 PMC5873268

[83] Kemény L Berggren L Dossenbach M Dutronc Y Paul C. Efficacy and safety of ixekizumab in patients with plaque psoriasis across different degrees of disease severity: results from UNCOVER-2 and UNCOVER-3. J Dermatol Treat. (2019) 30:19–26. 10.1080/09546634.2018.1473551 29726739

[84] Paul C Griffiths CEM van de Kerkhof PCM Puig L Dutronc Y Henneges C. Ixekizumab provides superior efficacy compared with ustekinumab over 52 weeks of treatment: results from IXORA-S, a phase 3 study. J Am Acad Dermatol. (2019) 80:70–9.e3. 10.1016/j.jaad.2018.06.039 29969700

[85] Gottlieb A Merola JF Psoriatic arthritis for dermatologists. J Dermatol Treat. (2019) 2019:1–18. 10.1080/09546634.2019.1605142 31014154

[86] ClinicalTrials.gov Study of Efficacy and Safety of Secukinumab in Participants With Active Psoriatic Arthritis With Axial Skeleton Involvement (MAXIMISE). Bethesda, MD: National Library of Medicine (US) (2000). Available online at: https://clinicaltrials.gov/ct2/show/NCT02721966 (accessed June 3, 2020).

[87] Fredriksson T Pettersson U. Severe psoriasis–oral therapy with a new retinoid. Dermatologica. (1978) 157:238–44. 10.1159/000250839 357213

[88] Elewski BE Baker CS Crowley JJ Poulin Y Okun MM Calimlim B. Adalimumab for nail psoriasis: efficacy and safety over 52 weeks from a phase-3, randomized, placebo-controlled trial. J Eur Acad Dermatol Venereol. (2019) 33:2168–78. 10.1111/jdv.15793 31304993 PMC6899987

[89] Ortonne JP Paul C Berardesca E Marino V Gallo G Brault Y. A 24-week randomized clinical trial investigating the efficacy and safety of two doses of etanercept in nail psoriasis. Br J Dermatol. (2013) 168:1080–7. 10.1111/bjd.12060 23013207

[90] Nikolakopoulou A Higgins JP Papakonstantinou T Chaimani A Del Giovane C Egger M Assessing confidence in the results of network meta-analysis (Cinema). bioRxiv. (2019) 2019:597047 10.1101/597047 PMC7122720 32243458

[91] Nikolakopoulou A Higgins JPT Papakonstantinou T Chaimani A Del Giovane C Egger M. CINeMA: an approach for assessing confidence in the results of a network meta-analysis. PLoS Med. (2020) 17:e1003082. 10.1371/journal.pmed.1003082 32243458 PMC7122720

[92] Papakonstantinou T Nikolakopoulou A Higgins JPT Egger M Salanti G CINeMA: software for semiautomated assessment of the confidence in the results of network meta-analysis. Campbell Syst Rev. (2020) 16:e1080 10.1002/cl2.1080 PMC8356302 37131978

[93] Mbuagbaw L Rochwerg B Jaeschke R Heels-Andsell D Alhazzani W Thabane L. Approaches to interpreting and choosing the best treatments in network meta-analyses. Syst Rev. (2017) 6:79. 10.1186/s13643-017-0473-z 28403893 PMC5389085

[94] Tada Y Watanabe R Noma H Kanai Y Nomura T Kaneko K. Short-term effectiveness of biologics in patients with moderate-to-severe plaque psoriasis: a systematic review and network meta-analysis. J Dermatol Sci. (2020) 99:53–61. 10.1016/j.jdermsci.2020.06.003 32600737

[95] Shi J Xu J Chen Y. A network meta-analysis for the comparison of efficacy and safety of interleukin (IL)-23 targeted drugs in the treatment of moderate to severe psoriasis. Dermatol Ther. (2020) 2020:e13802. 10.1111/dth.13802 32521069

[96] Augustin M Wirth D Mahlich J Pepper AN Druchok C. Cost per responder analysis of guselkumab versus targeted therapies in the treatment of moderate to severe plaque psoriasis in Germany. J Dermatol Treat. (2020) 2020:1–7. 10.1080/09546634.2020.1793891 32663067

[97] Kawalec P Holko P Moćko P Pilc A. Comparative effectiveness of abatacept, apremilast, secukinumab and ustekinumab treatment of psoriatic arthritis: a systematic review and network meta-analysis. Rheumatol Int. (2018) 38:189–201. 10.1007/s00296-017-3919-7 29285605 PMC5773655

[98] McInnes IB Nash P Ritchlin C Choy EH Kanters S Thom H. Secukinumab for psoriatic arthritis: comparative effectiveness versus licensed biologics/apremilast: a network meta-analysis. J Comp Effect Res. (2018) 7:1107–23. 10.2217/cer-2018-0075 30230361

[99] Song GG Lee YH. Relative efficacy and safety of apremilast, secukinumab, and ustekinumab for the treatment of psoriatic arthritis. Zeitschr Rheumatol. (2018) 77:613–20. 10.1007/s00393-017-0355-8 28791450

[100] Strand V Elaine Husni M Betts KA Song Y Singh R Griffith J. Network meta-analysis and cost per responder of targeted immunomodulators in the treatment of active psoriatic arthritis. BMC Rheumatol. (2018) 2:3. 10.1186/s41927-018-0011-1 30886954 PMC6390550

[101] Ruyssen-Witrand A Perry R Watkins C Braileanu G Kumar G Kiri S. Efficacy and safety of biologics in psoriatic arthritis: a systematic literature review and network meta-analysis. RMD Open. (2020) 6:e001117. 10.1136/rmdopen-2019-001117 32094304 PMC7046955

[102] Wervers K Luime JJ Tchetverikov I Gerards AH Kok MR Appels CWY. Influence of disease manifestations on health-related quality of life in early psoriatic arthritis. J Rheumatol. (2018) 45:1526–31. 10.3899/jrheum.171406 29961685

[103] Sunar I Ataman S Nas K Kilic E Sargin B Kasman SA. Enthesitis and its relationship with disease activity, functional status, and quality of life in psoriatic arthritis: a multi-center study. Rheumatol Int. (2020) 40:283–94. 10.1007/s00296-019-04480-9 31773391

[104] Kristensen S Christensen JH Schmidt EB Olesen JL Johansen MB Arvesen KB. Assessment of enthesitis in patients with psoriatic arthritis using clinical examination and ultrasound. Muscles Ligaments Tendons J. (2016) 6:241–7. 10.11138/mltj/2016.6.2.241 27900299 PMC5115257

[105] Palominos PE de Campos APB Ribeiro SLE Xavier RM Xavier JW de Oliveira FB. Correlation of enthesitis indices with disease activity and function in axial and peripheral spondyloarthritis: a cross-sectional study comparing MASES, SPARCC and LEI. Adv Rheumatol. (2019) 59:23. 10.1186/s42358-019-0066-8 31208465

[106] Mease PJ Van den Bosch F Sieper J Xia Y Pangan AL Song IH. Performance of 3 Enthesitis indices in patients with peripheral spondyloarthritis during treatment with adalimumab. J Rheumatol. (2017) 44:599–608. 10.3899/jrheum.160387 28298558

[107] Vieira-Sousa E Alves P Dactylitis: more than just arthritis. Acta Reumatol Portuguesa. (2015) 40:210–2. 26535776

[108] Kaeley GS Eder L Aydin SZ Gutierrez M Bakewell C. Dactylitis: a hallmark of psoriatic arthritis. Semin Arthr Rheumat. (2018) 48:263–73. 10.1016/j.semarthrit.2018.02.002 29573849

[109] Walsh J Ogdie A Michaud K Peterson S Holdsworth E Karyekar C Enthesitis, Dactylitis, and Axial Disease in Psoriatic Arthritis (psa): Impact on Patient Quality of Life and Work Productivity. (2019). Available online at: https://acrabstracts.org/abstract/enthesitis-dactylitis-and-axial-disease-in-psoriatic-arthritis-psa-impact-on-patient-quality-of-life-and-work-productivity/ (accessed August 14, 2020).

[110] Chandran V Tolusso DC Cook RJ Gladman DD. Risk factors for axial inflammatory arthritis in patients with psoriatic arthritis. J Rheumatol. (2010) 37:809–15. 10.3899/jrheum.091059 20231209

[111] Feld J Chandran V Haroon N Inman R Gladman D. Axial disease in psoriatic arthritis and ankylosing spondylitis: a critical comparison. Nat Rev Rheumatol. (2018) 14:363–71. 10.1038/s41584-018-0006-8 29752461

[112] Nash P Lubrano E Cauli A Taylor WJ Olivieri I Gladman DD. Updated guidelines for the management of axial disease in psoriatic arthritis. J Rheumatol. (2014) 41:2286–9. 10.3899/jrheum.140877 25362712

[113] Lubrano E Parsons WJ Perrotta FM. Assessment of response to treatment, remission, and minimal disease activity in axial psoriatic arthritis treated with tumor necrosis factor inhibitors. J Rheumatol. (2016) 43:918–23. 10.3899/jrheum.151404 26980581

[114] Nagayasu A Nawata M Saito K Tanaka Y. Short-term effectiveness of ixekizumab to refractory psoriatic arthritis with spondyloarthritis: two case reports. Modern Rheumatol Case Rep. (2020) 4:176–80. 10.1080/24725625.2019.1703546 33086996

[115] Kavanaugh A Puig L Gottlieb AB Ritchlin C You Y Li S. Efficacy and safety of ustekinumab in psoriatic arthritis patients with peripheral arthritis and physician-reported spondylitis: *post*-*hoc* analyses from two phase III, multicentre, double-blind, placebo-controlled studies (PSUMMIT-1/PSUMMIT-2). Ann Rheum Dis. (2016) 75:1984–8. 10.1136/annrheumdis-2015-209068 27098404

[116] Baraliakos X Gossec L Pournara E Jeka S Blanco R D'Angelo S Secukinumab Provides Sustained Improvements in Clinical and Imaging Outcomes in Patients With Psoriatic Arthritis and Axial Manifestations: Results From the MAXIMISE Trial. (2020). Available online at: https://acrabstracts.org/abstract/secukinumab-provides-sustained-improvements-in-clinical-and-imaging-outcomes-in-patients-with-psoriatic-arthritis-and-axial-manifestations-results-from-the-maximise-trial/ (accessed November 13, 2020).

[117] Raposo I Torres T. Nail psoriasis as a predictor of the development of psoriatic arthritis. Actas Dermo Sifiliograficas. (2015) 106:452–7. 10.1016/j.ad.2015.02.005 26026773

[118] Wu D Yue J Tam LS Efficacy and safety of biologics targeting interleukin-6,-12/23 and-17 pathways for peripheral psoriatic arthritis: a network meta-analysis. Rheumatology. (2018) 57:563–71. 10.1093/rheumatology/kex452 29244162

